# Natural Contrast Statistics Facilitate Human Face Categorization

**DOI:** 10.1523/ENEURO.0420-21.2022

**Published:** 2022-10-04

**Authors:** Joan Liu-Shuang, Yu-Fang Yang, Bruno Rossion, Valérie Goffaux

**Affiliations:** 1 Institute of Research in Psychology (IPSY), University of Louvain, Louvain-la-Neuve 1348, Belgium; 2 Institute of Neuroscience (IoNS), University of Louvain, Louvain-la-Neuve 1348, Belgium; 3 Université de Lorraine, Centre National de la Recherche Scientifique, Nancy CRAN F-54000, France; 4 Service de Neurologie, Université de Lorraine, Centre Hospitalier Régional Universitaire (CHRU)-Nancy, Nancy F-54000, France; 5 Department of Cognitive Neuroscience, Maastricht University, Maastricht 6229, The Netherlands

**Keywords:** contrast polarity, EEG, face categorization, frequency tagging, natural statistics

## Abstract

The ability to detect faces in the environment is of utmost ecological importance for human social adaptation. While face categorization is efficient, fast and robust to sensory degradation, it is massively impaired when the facial stimulus does not match the natural contrast statistics of this visual category, i.e., the typically experienced ordered alternation of relatively darker and lighter regions of the face. To clarify this phenomenon, we characterized the contribution of natural contrast statistics to face categorization. Specifically, 31 human adults viewed various natural images of nonface categories at a rate of 12 Hz, with highly variable images of faces occurring every eight stimuli (1.5 Hz). As in previous studies, neural responses at 1.5 Hz as measured with high-density electroencephalography (EEG) provided an objective neural index of face categorization. Here, when face images were shown in their naturally experienced contrast statistics, the 1.5-Hz face categorization response emerged over occipito-temporal electrodes at very low contrast [5.1%, or 0.009 root-mean-square (RMS) contrast], quickly reaching optimal amplitude at 22.6% of contrast (i.e., RMS contrast of 0.041). Despite contrast negation preserving an image’s spectral and geometrical properties, negative contrast images required twice as much contrast to trigger a face categorization response, and three times as much to reach optimum. These observations characterize how the internally stored natural contrast statistics of the face category facilitate visual processing for the sake of fast and efficient face categorization.

## Significance Statement

Human faces share a universal property: the strict alternation of contrast, with the darker main features against the more uniform, lighter skin. The ability to categorize faces depends critically on the presence of these natural contrast statistics in the input stimulus. However, it is not yet known how natural contrast statistics facilitate the visual processing leading to categorization. Using frequency tagging and high-density electroencephalography, we show that access to internally stored natural statistics reduces the amount of sensory input necessary for human face categorization.

## Introduction

**Figure 1.**
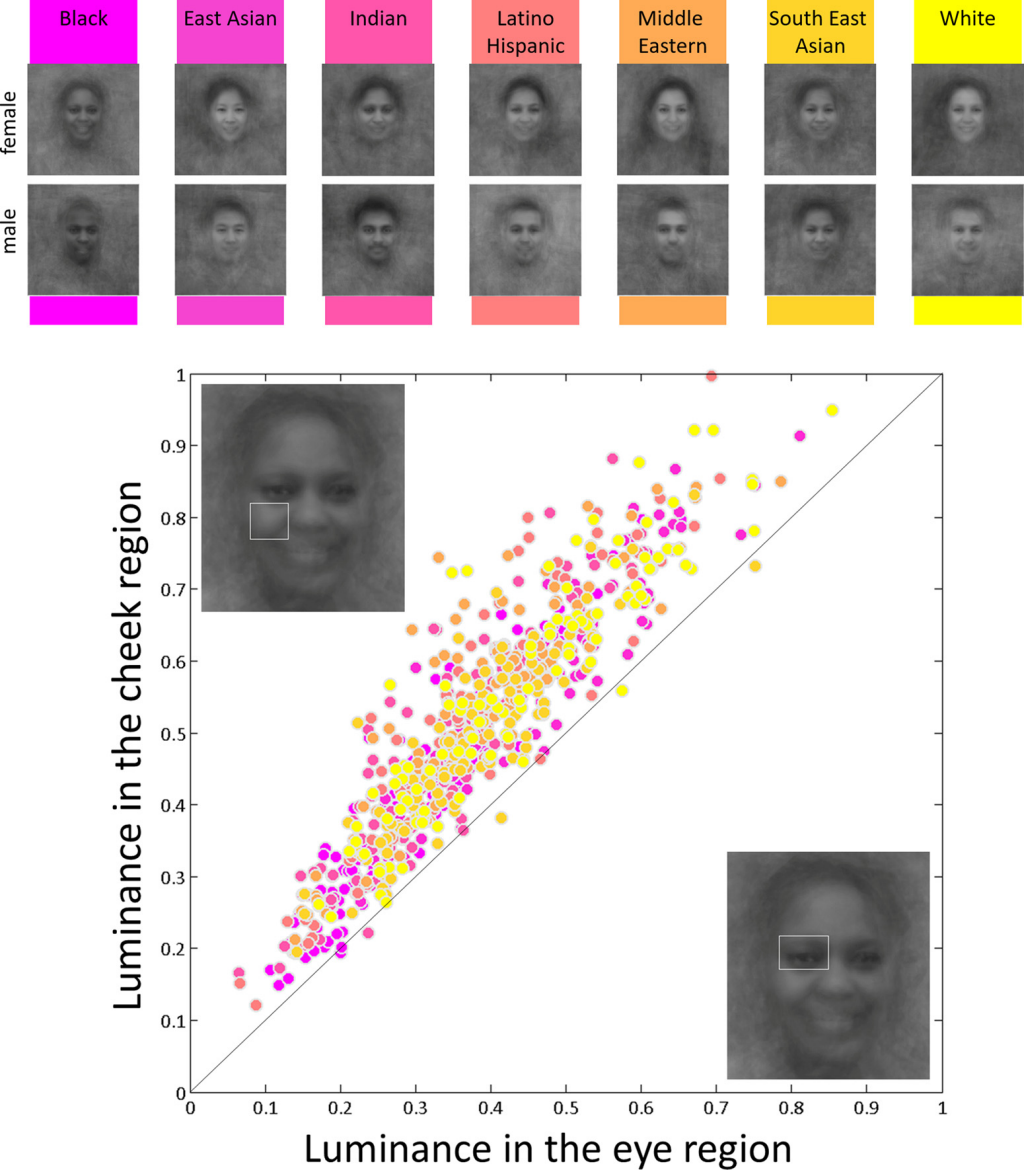
The human face obeys systematic and universal contrast statistics with the regions occupied by the main features being darker than the neighboring skin surface. To illustrate this point, we measured the luminance in the left eye and left cheek regions in a large set of “in-the-wild” face images stemming from seven distinct ethnic origins (*n* = 100 images per ethnicity, half-female; age range: 30–39 years old; Karkkainen and Joo, 2021). We used this image set for the luminance measurements exclusively (i.e., a different, validated, set of faces was used as experimental stimuli). We only used face images where these regions were free of hair, occlusion (by e.g., glasses, or a limb), and excessive make-up. Faces were of highly variable pose, expression, illumination, etc. The broad distribution of luminance values illustrates the large heterogeneity of skin tones measured. Top, Illustrative averages of the face images that were used for the analysis in each ethnic origin. Bottom, Scatterplot of the mean luminance of the eye and the cheek region for each measured faces (*n* = 700 in total; for a similar analysis on a restricted set of 50 faces, see Gilad et al., 2009). White rectangles superimposed on the inset faces illustrate the eye and cheek regions as sampled in the present analysis. Except for a minority of outlier face images, one can see that all data points lie above the diagonal, which means that luminance in the eye region is systematically darker than around the cheek. This rule applies regardless of face surface properties (absorbance, illumination, and specular reflection).

Among the many things that humans encounter in their visual environment, there is one category of utmost ecological relevance, and for which the human brain has developed an exceptionally high sensitivity: the faces of conspecifics. The newborn human preferentially orients their gaze toward faces and face-like patterns (Goren et al., 1975; Turati et al., 2002; Buiatti et al., 2019) and infants of a few months of age already show specialized (right-lateralized) neural responses to faces presented at a glance (de Heering and Rossion, 2015; Rekow et al., 2021; see also Tzourio-Mazoyer et al., 2002). At adulthood, humans automatically detect the presence of a face in the environment, and differentiate it from the other objects composing the scene (i.e., categorize it as a face) at presentation duration as short as 17 ms (Keysers et al., 2001; Retter et al., 2020; see also Grill-Spector and Kanwisher, 2005; Fisch et al., 2009; Perrett et al., 2009; Mohsenzadeh et al., 2018). It only takes a few tens of milliseconds to initiate a saccade toward a face even if it is embedded in a complex background (Thorpe et al., 1996; Lewis and Edmonds, 2003; Rousselet et al., 2003; Hershler and Hochstein, 2005; Fletcher-Watson et al., 2008; Honey et al., 2008; Crouzet et al., 2010; Crouzet and Thorpe, 2011; Martin et al., 2018; Kauffmann et al., 2019; for review, see Fabre-Thorpe, 2011). The mechanisms underlying the automaticity and exceptional speed at which human adults categorize faces work efficiently even when access to sensory input is hampered because of brief exposure, masking, blur, noise, or clutter (Goffaux et al., 2011; Ales et al., 2012; Quek et al., 2018b). Yet, face categorization is dramatically impaired when the stimulus does not share the visual properties that have been experienced by the observer as being characteristic of the face category (Rosch and Mervis, 1975; Rosch et al., 1976), i.e., the regularities in how faces appear in everyday life (so-called natural statistics; Geisler, 2008; Clifford et al., 2015). One salient and universal regularity of the human face is the ordered alternation of contrast, with darker regions occupied by the main features (brows, eyes, nose and mouth) against the more uniform and relatively lighter skin surface (Fig. 1; Watt, 1994; Dakin and Watt, 2009; Gilad et al., 2009). This relative distribution of contrast is because of the structural and reflectance properties of the face and the fact that lighting usually comes from above (Ramachandran, 1988; Liu et al., 1999). Such characteristic natural light/dark alternation is reversed with image negation. Although negation is a fully reversible manipulation of the image, which leaves the image geometric and spectral properties unchanged, it has profound consequences for the categorization of faces.

**Figure 2.**
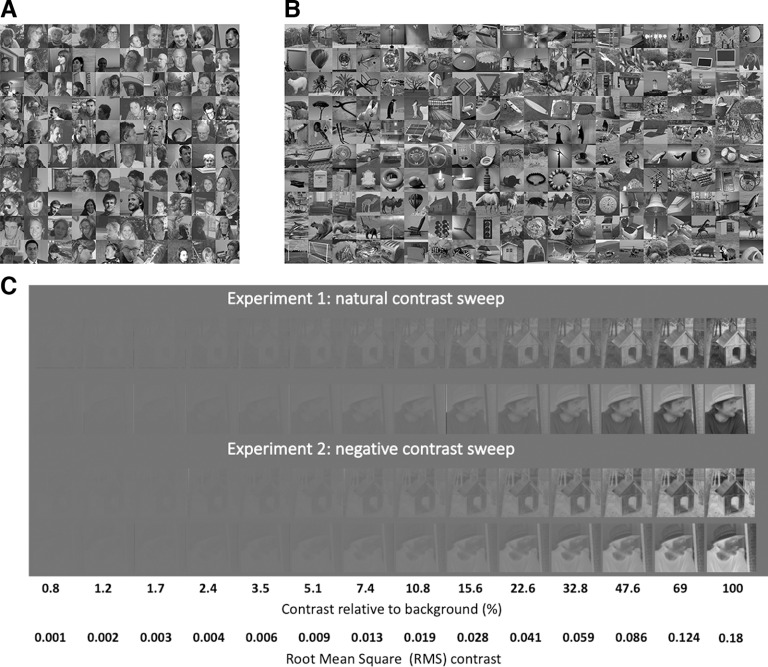
Experimental stimuli. ***A***, Face images (*N* = 100). ***B***, Nonface images (*N* = 200). ***C***, Stimulus contrast was modulated via α blending (i.e., luminance values were calculated as a weighted average of the stimulus with the gray background). Example object and face image at the different contrast levels, in natural and negative contrast groups. Note that luminance and contrast were here adjusted for figure visibility and are not representative of the actual (γ-corrected) values.

For example, contrast negation disrupts human infants’ viewing preference for faces (Farroni et al., 2005; Otsuka et al., 2012). Later in adulthood, contrast negation slows down and impairs behavioral performance in face detection (Lewis and Edmonds, 2003; Liu-Shuang et al., 2015a) as well as face identity recognition tasks (Galper, 1970; Galper and Hochberg, 1971; Bruce and Langton, 1994; Kemp et al., 1996; Liu et al., 1999; Lewis and Edmonds, 2003; Russell et al., 2006; Nederhouser et al., 2007; Garrido et al., 2008; Liu-Shuang et al., 2015a). This manipulation also reduces the human brain response to faces (George et al., 1999; White, 2001; Vuong et al., 2005; Nederhouser et al., 2007; Gilad et al., 2009; Nasr and Tootell, 2012; Rossion et al., 2012; Yue et al., 2013; Liu-Shuang et al., 2015a; see also Ohayon et al., 2012 for evidence in the primate brain). Altogether, these findings suggest that human efficiency at categorizing faces critically depends on whether their appearance obeys the naturally experienced statistics of the face category, i.e., whether it depicts the contrast pattern typically rendered by a top-lit face. Comparatively, contrast negation has a moderate impact on the categorization of nonface stimuli (Galper, 1970; Nederhouser et al., 2007; Yue et al., 2013), which has been taken to suggest that the unique susceptibility of human face categorization to contrast negation is mainly because of (1) face appearance obeying unique universal ordinal contrast rules (Fig. 1), and (2) their extensive learning by humans over lifespan.

In studies addressing the influence of contrast polarity statistics on face categorization, the stimuli are typically a few homogeneous exemplars isolated from their natural context and selected from a restricted set of categories (Yue et al., 2013; Liu-Shuang et al., 2015a). However, in real life, categorization success depends on the dual ability to differentiate faces from their background (i.e., segmentation) and from diverse distracters that are present in the scene as well as to generalize across widely variable, and sometimes very dissimilar, category exemplars (for review, see Rossion et al., 2018). Such naturalistic, i.e., variable and crowded, viewing conditions, place maximum demands on the sensitivity and tolerance of face categorization, and it is in such viewing conditions that the knowledge of natural statistics is expected to contribute the most to perception (Clifford et al., 2015; Karimi-Rouzbahani et al., 2021). In order to adequately define the influence of natural statistics on the genuine categorization of faces, it is therefore crucial to simulate the richness of real-world visual diet and, therefore, to use naturalistic, heterogeneous, and unsegmented face stimuli contrasted to a wide array of biological and man-made objects.

As noted above, while a well-known signature of human face recognition is its right hemispheric dominance, the causes/factors of this right-hemispheric lateralization remain a mystery (Behrmann and Plaut, 2020; Rossion and Lochy, 2022). When sensory input is reduced, e.g., by poor visual acuity during early development or with stimulus degradation, the right-hemispheric dominance is preserved, even enhanced (de Schonen and Mathivet, 1990; Quek et al., 2018b). Since contrast negation breaks the natural contrast statistics of the human face that are at the heart of the neural specialization of face recognition, their preservation may be necessary to observe a right-hemispheric dominance. If this reasoning is correct, the right-hemispheric dominance of human face categorization should disappear, or at least be significantly reduced, with contrast negation (cf. Liu-Shuang et al., 2015a).

Another fundamental issue is exactly how natural statistics support the successful categorization of faces. Past evidence has shown that contrast negation weakens neural and behavioral responses to faces but exactly how it impedes visual processing leading up to (genuine) face categorization is unclear.

**Figure 3.**
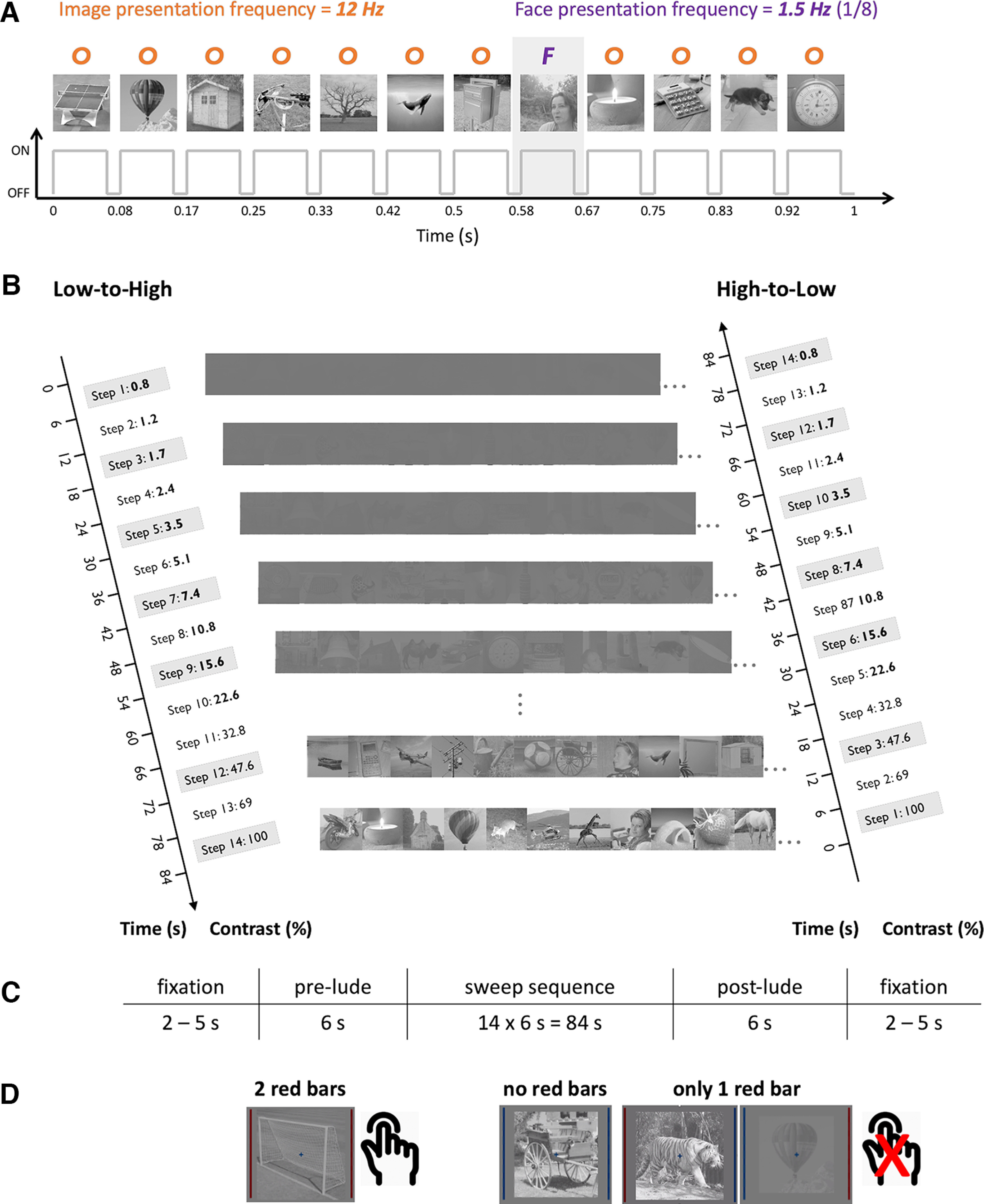
***A***, EEG frequency-tagging paradigm. Images were presented in random order at a rate of 12 Hz (12 images/s, SOA of 83.33 ms), with faces embedded among objects at fixed intervals of 1/8 (face presentation frequency = 12/8 = 1.5 Hz). ***B***, The image contrast of the frequency-tagged sequences was parametrically varied over 14 × 6 s steps (84 s total), either gradually increasing (Low-to-High, left) or decreasing (High-to-Low, right). Sweeping in the two directions enabled to account for potential order effects on responses. ***C***, Schematic timeline of a sweep trial. Following the appearance of a central fixation cross (lasting between 2 and 5 s), the sequence began with a pre-lude period (repetition of the first sweep step) before the sequence proper. At the end of the sequence, the last sweep step was repeated during the post-lude period, and the fixation remained on screen for another 2–5 s. ***D***, Observers performed an orthogonal behavioral vigilance task during which they maintained central fixation while monitoring two flanking vertical bars. These bars independently changed color from red to blue and observers had to respond only when both bars turned red simultaneously. Extended Data Fig. 3-1 illustrates the group-averaged accuracy and reaction times in each group and condition. Note that luminance and contrast were here adjusted for figure visibility and are not representative of the actual (gamma-corrected) values.

In order to clarify these outstanding issues, the present study elicited genuine visual categorization processes by using a large and variable set of face and nonface (manmade objects, buildings, plants, animals) exemplars embedded in their original context. The depicted faces varied substantially in age, gender, expression, pose, size and viewpoint (Fig. 2*A*,*B*). This wide variety of images places the visual system in a mode of operation closer to that activated in the natural conditions of face categorization, and necessarily engages its sensitivity and tolerance. We present these images at a fast periodic rate of 12 Hz [stimulus onset asynchrony (SOA) of 83.33 ms], which is optimal for categorization in these conditions (Retter et al., 2020), and record human brain responses using high-density scalp electroencephalography (EEG). Since the human brain synchronizes its activity to the periodicity of the visual stimulus (Adrian and Matthews, 1934; Regan, 1966; for review, see Norcia et al., 2015), such a stimulus sequence elicits a common neural response to face and nonface stimuli at image frequency rate (12 Hz) and harmonics. Face stimuli appeared every 8th image (i.e., periodicity of 1.5 Hz; Fig. 3*A*; Extended data Figure 3-1). If the periodically presented faces are successfully categorized, an additional category-selective neural response emerges at 1.5 Hz, i.e., at the same frequency as face occurrence.

10.1523/ENEURO.0420-21.2022.f3-1 Extended Data Figure 3-1 Group-averaged accuracy and correct reaction times in the vigilance task during the sweep and full-contrast sequences, separately. ***A***. Behavioral performance in the natural contrast group. ***B***. Behavioral performance in the negative contrast group. These histograms show the means accuracy for each presentation mode. Secondary y-axis presents mean reaction time values (millisecond). Error bars represent 95% confidence intervals. Note that luminance and contrast were adjusted for figure visibility and are not representative of the actual (gamma-corrected) values. Download Figure 3-1, TIF file.

This neural response at 1.5 Hz is an implicit marker of genuine categorization, i.e., one that meets the dual requirements of face categorization: sensitivity to the face relative to the other categories present in each scene and sequence, as well as tolerance to the high variability of face appearance in the sequence (Rossion et al., 2015, 2018; Retter and Rossion, 2016; Peykarjou et al., 2017; Quek and Rossion, 2017; Gao et al., 2018, 2019; Retter et al., 2018; Or et al., 2019; Hauk et al., 2021). Its amplitude has recently been shown to scale systematically with the proportion of face images that are correctly categorized in a sequence (Retter et al., 2020). This implicit frequency-tagged marker of face categorization usually emerges over occipito-temporal scalp electrodes, mainly right-lateralized, and presumably reflect face-selective responses in the ventral occipito-temporal cortex (see Jonas et al. (2016) for evidence from human intracerebral recordings, Gao et al. (2018) for functional magnetic resonance imaging evidence, and Hauk et al. (2021) with magnetoencephalography). A categorization response also emerges when nonface categories are frequency-tagged in fast stimulation sequences (e.g., houses and body parts Jonas et al., 2016; Hagen et al., 2020). However, it is of much weaker amplitude for nonface than for face categorization and associated with distinct patterns of lateralization and scalp topography. This paradigm has multiple advantages over other approaches. Not only does it lead to a more authentic categorization, but it also measures the brain’s response to categorization implicitly and objectively, at time frequencies predetermined (“labeled”) by the periodicity of the sequence (Retter and Rossion, 2016).

Here we used this well-validated approach to characterize how natural face contrast statistics affects the face categorization process. We compared the neural face categorization response as a function of sensory input, for image sequences displayed in a natural contrast polarity, to sequences with reversed contrast polarity (negative contrast). In each sequence, stimulus contrast ramped (i.e., was “swept”) up or down (Fig. 3*B*). Contrast being the fundamental signal driving the visual system (Kaplan et al., 1987; Boynton et al., 1996), its sweeping enables manipulating the amount of sensory input available to categorize faces. With this approach, we defined for each individual participant the minimal amount of sensory input (i.e., contrast) required to elicit a genuine categorization response as well as the optimal level of sensory input, namely the level of contrast needed to see a full-blown categorization response, i.e., reaching the amplitude as elicited by full contrast images.

We reasoned that the facilitation of categorization by natural statistics may take multiple forms. Humans may simply need less sensory input to start categorizing a face as a face. In this case, contrast negation should only increase the contrast minimum and leave the optimum level of contrast unchanged. It is however plausible that once the minimal amount of information has been reached and leads to a significant categorization response, the visual system may need less input to accrue the face categorization response until its optimum (Liu-Shuang et al., 2015a; Quek et al., 2018b; Retter et al., 2020). By deriving the distance separating minimum/optimum, we can define the dynamics of visual processing subtending face categorization. An increase in the contrast minimum for contrast-negated sequences will reduce such distance while an increase of optimum will enlarge the minimum/optimum distance. This approach allows us to characterize how the dynamics of visual processing subtending face categorization are modulated by natural face contrast statistics.

## Materials and Methods

### Participants

Thirty-one healthy adult participants were recruited online through the university’s participation pool in exchange for monetary compensation (10 €/h). Sixteen participants viewed stimulus sequences in their natural contrast polarity (“natural contrast” group). We excluded one individual whose EEG recording contained excessive artifact noise (deflections exceeding ±100 μV on multiple epochs). The final “natural contrast” sample consisted of 15 participants (nine females, age = 23 ± 3 years). Another, so-called “negative contrast” group of 15 participants (six females, age = 23 ± 1.7 years) was tested with the contrast-negated stimulus sequences. We opted for a between-subject manipulation of contrast polarity to avoid any potential learning/order effects. All observers were right-handed as confirmed by an electronic version of the Edinburgh Handedness Inventory measurement (Oldfield, 1971) and reported normal or corrected-to-normal vision. Participants gave written informed consent prior testing conformingly to the guidelines of the local Biomedical Ethical committee (B403201111965).

### Stimuli

Stimuli were 256 × 256 pixel greyscale images of 100 white faces and a variety of 200 nonface items (e.g., animals, plants, man-made objects, buildings, etc.). All faces and objects were embedded in their natural backgrounds (i.e., unsegmented), and varied greatly in orientation, lighting, size, and overall appearance (Fig. 2*A*,*B*). The same stimulus set was used in previous studies (Quek and Rossion, 2017; Quek et al., 2018b). For the present study specifically, we equalized mean luminance and contrast of all the stimuli by first normalizing the pixel luminance to a mean of zero and a SD [i.e., root-mean-square (RMS) contrast] of one. Face and object images were contrast-negated by flipping the pixel intensities around the mean luminance value (Fig. 2*C*). Contrast negation was used here to disrupt the natural statistics of the images thought to crucially support their visual processing while conserving their amplitude spectrum and contours (see below). All images were attributed a global luminance of 0.46 and an RMS contrast of 0.18. These values reflect the grand-averaged luminance and contrast values of the original images, adjusted to avoid luminance clipping. The resulting face and object images showed similar average pixel luminance distributions.

Finally, images were γ-corrected according to the luminance profile of the BenQ XL2420T monitor (refresh rate of 120 Hz, screen resolution of 1920 × 1080 pixels). The mean luminance after γ correction was 58.79 cd/m^2^. Stimulation was conducted on Java SE version 8 in Windows 7. Stimuli were displayed at a viewing distance of 100 cm and subtended a visual angle of 4.04° by 4.04° in a dimly lit room. During stimulation, image contrast was parametrically manipulated on a logarithmic scale via online α blending, which created a weighted sum of the luminance values of the image and a gray background while maintaining mean luminance. Fourteen contrast levels were presented, from 0.8% to 100% of the initial RMS contrast, with percentages referring to the weight of images relative to background. The respective RMS contrast values of the presented stimuli are listed on Figure 2. Logarithmic scaling was chosen to maximize sampling of the low contrast range where the human visual system is expected to be most sensitive.

### Procedure

Before each EEG experiment, we measured the visual abilities of each participant using the Freiburg Visual Acuity and Contrast Test (FrACT; Landolt C optotype stimulus with 4AFC) at a viewing distance of 100 cm. Mean minimums in terms of Weber contrast were 1.02% for the “natural contrast” group (range = 0.6–1.67%) and 1.05% in the “negative contrast” group (range = 0.59–2.18%), and did not differ significantly between groups (independent samples *t* test: *t*_(28)_ = −0.21, *p* = 0.84).

In an experimental sequence, images were shown at a fast rate of 12 Hz (12 images/s, SOA = 83.33 ms) through a squarewave luminance modulation with an 80% duty cycle (i.e., images were ON for 66.67 ms and OFF for 16.666 ms; Fig. 3). Faces (F) were inserted among objects (O) at regular fixed intervals of 1/8 (or 12/8 = 1.5 Hz), resulting in the following stimulation pattern: OOOOOOOFOOOOOOOFOO… (Fig. 3*A*, as in Quek et al., 2018b). We opted for a 12-Hz periodicity because of consistent evidence that this rate elicits the largest EEG categorization responses as well near-ceiling behavioral performance (Retter et al., 2020). In order to minimize potential low-level edge and image adaptation effects, at each 12-Hz presentation cycle, stimulus size randomly varied between 90% and 110% (in 5% steps; i.e., five possible sizes). In agreement with previous studies (Quek et al., 2018b), we expected this periodic stimulation to elicit two critical neural responses: (1) a general visual response at the 12-Hz image presentation frequency (and harmonics), reflecting general visual processing common to faces and objects; and (2) a face categorization response at the 1.5-Hz face presentation frequency (and harmonics). Considering that our study addresses the impact of natural statistics on categorization efficiency, the subsequent analyses will mainly focus on the face categorization response.

The main experiment employed so-called contrast sweep sequences (Fig. 3*B*), in which we varied image contrast over 14 sequential steps (6 s per step), either in ascending (low to high) or descending order (high to low). Contrast was swept in both directions to compensate for potential order effects. Hence, image sequences either gradually appeared or gradually disappeared against the gray background over the course of each 84-s sweep sequence. At the face presentation rate of 1.5 Hz, each step contained 9 faces. A sequence was flanked by six extra seconds consisting, respectively, of repetitions of the first contrast step (e.g., 0.8% in the Low-to-High condition) and the last contrast step (e.g., 100% in the Low-to-High condition). As in previous studies (Quek et al., 2018b), these “pre-lude” and “post-lude” periods, aiming to minimize muscle artefacts and ocular movement elicited by the appearance and disappearance of flickering images, were not further analyzed. We presented a total of 24 sequences (half in Low-to-High order and the other half in High-to-Low order), divided into four blocks of 6 sequences shown in pseudorandomized order. With nine face images presented per sequence step, we reached a total of 3024 face image presentations in the main experiment (and a total of 21,168 nonface images).

Tracking the amplitude of the 12-Hz general visual response and of the 1.5-Hz face categorization response across contrast steps enabled us to define the minimum and optimum levels of contrast for each neural response (see below).

The main experiment was followed by one block of four 96-s (6 + 84 + 6 s as for contrast sweep sequences) long full-contrast sequences of natural contrast images, in which image contrast remained at 100% without modulation, except for the pre-lude and post-lude. These latter sequences provided reference amplitudes for EEG responses recorded in the contrast sweep sequences. In the “negative contrast” group of participants, we additionally presented four full-contrast sequences with negative contrast. Importantly, the response profile of each subject was evaluated against its own full positive contrast reference response, i.e., minima and optima were defined within subject.

### Behavioral task and performance

A blue central fixation cross (0.05 × 0.2° visual angle) was overlaid on top of the stimulation sequence, appearing 2–5 s before the onset of the sequence and remaining 2–5 s after its offset. Two vertical lateral bars (visual angle 0.08 × 4.3°) flanked the stimulus continuously at an eccentricity of 2.02°. In the beginning the trial, the both vertical bars were blue. At 12 (pseudo)random latencies within the 84-s sweep sequence, either one of the vertical bars or both of the vertical bars turned red for a duration of 300 ms. At all other times during the sequence, both bars were shown as blue. Each change lasted for 300 ms, and there was a minimum interval of 800 ms between consecutive changes. The interval between the offset of the red bar(s) and the onset of the next red bar(s), that is, the time during which both bars were blue, was always at least 800 ms. Participants were instructed to maintain central fixation and to respond whenever both bars simultaneously changed color by pressing on the spacebar of a keyboard. On average, there were ∼6 targets per sequence (mean ± SD: natural contrast group = 5.92 ± 1.74; negative contrast group = 5.98 ± 1.69). Responses were considered correct when falling within a time-window between 250 and 800 ms following color change onset.

Previous studies have shown that the neural response to frequency-tagged face categorization is robust whether participants perform this task, another vigilance task or no task at all (Quek et al., 2018a,b). In the present study, a vigilance task was required to ensure that participants’ attention to the stimulus was comparable across contrast levels and groups. Overall, behavioral performance in the full contrast and contrast sweep conditions was highly accurate across groups (Extended Data Fig. 3-1). Independent samples *t* tests did not reveal any Group differences in accuracy, neither in the Full contrast (*t*_(28)_ = −0.052, *p* = 0.96, 95% confidence interval of the group difference: [–0.078 0.074]), nor in the contrast sweep sequences (*t*_(28)_ = 0.4, *p* = 0.69, 95% confidence interval of the group difference: [–0.036 0.054]). Mean correct response times were also similar across Groups (Full contrast sequence: *t*_(28)_ = −0.86, *p* = 0.4, 95% confidence interval of the group difference: [–0.069 0.028]; Contrast sweep sequence: *t*_(28)_ = –0.54, *p* = 0.6, 95% confidence interval of the group difference: [–0.054 0.031]). Overall, these findings suggest that both groups were adequately and equally attentive to the stimulation sequences.

### EEG acquisition

Scalp EEG was recorded with 128 Ag-AgCl active-electrodes from the Biosemi ActiveTwo system (BioSemi B.V.) in a quiet and dimly-lit room. Default electrode labels (e.g., A1, B1, etc.) were renamed to approximate the 10/5 system (Oostenveld and Praamstra, 2001; Rossion et al., 2015). The vertical and horizontal electrooculogram (EOG) was monitored with four additional flat-type electrodes placed at the outer canthi of the eyes, and above and below the right eye. EEG data were sampled at 512 Hz, and the magnitude of electrode offset was held below ±25 mV.

### Data analysis

Natural and negative contrast experiments followed the same analysis procedure outlined below.

#### Preprocessing

EEG data analysis was performed using Letswave 5 (https://www.letswave.org/) running on MATLAB R2012b (MathWorks). We first de-trended and removed the DC component from the continuous EEG signal before applying a 0.1- to 120-Hz bandpass filter (4th order zero-phase Butterworth filter). Data were downsampled to 256 Hz for easier handling and segmented into sequences according to condition (High-to-Low contrast sweep, Low-to-High contrast sweep, and full-contrast), with two extra seconds before and after each sequence (2 + 96 + 2 s = 100 s). For each participant, we corrected artifacts related to eye blinks by applying independent component analysis (ICA) with a square mixing matrix on each participant and removing the single component corresponding to the blinks, identified based on the component waveform and scalp topography. Further artifact-ridden channels (i.e., sudden amplitude shifts exceeding ±100 μV over several epochs) were replaced by the average of the three neighboring channels using linear interpolation. Next, the cleaned data were re-referenced to the average of the 128 scalp channels and averaged within conditions for each participant.

#### Frequency-domain analysis

In the sweep conditions, each preprocessed sequence was further cropped according to the 14 contrast steps into 6-s epochs, discarding the first and last 6 s corresponding to the pre-lude and post-lude. A fast Fourier transform (FFT) was applied to extract the frequency amplitude spectra for each step [i.e., normalized amplitude spectrum (μV), with a frequency range of 0–128 Hz and frequency resolution of 0.167 Hz]. For full-contrast sequences, we also cropped the four preprocessed sequences into 14 smaller epochs (total of 56 epochs) to obtain amplitude spectra with comparable frequency resolution as the sweep sequences. As there is no contrast modulation during these 14 epochs for full-contrast sequence, they were averaged together following the FFT.

**Figure 4.**
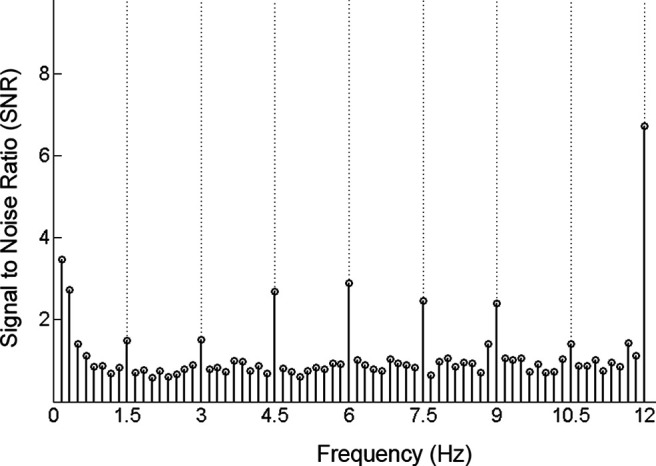
Frequency domain characterization of the neural response. Grand averaged SNR spectrum of the neural response to the contrast sweep at maximum visibility (i.e., step 14) at occipito-temporal channel (PO10). Note the robust response at the image presentation frequency (12 Hz and harmonics up to 48 Hz; not visible here). Responses to faces were tagged at 1.5 Hz and harmonics (1.5, 3, 4.5, 6, 7.5, 9, and 10.5 Hz).

To correct for variation in baseline noise level, the raw amplitudes were normalized relative to the background EEG activity by subtracting from the amplitude at the frequencies of interest (i.e., 1.5 Hz and its harmonics for face categorization responses, and the stimulus presentation rate of 12 Hz and its harmonics; Fig. 4) the mean amplitude of eight neighboring frequency bins (four on either side, skipping the immediately adjacent bin) in each condition and for each subject. To quantify general visual responses and face categorization responses, we aggregated across their respective frequency harmonics (similarly to Quek et al., 2018b). The range of harmonics was defined a priori based on previous experiments using the current face categorization frequency-tagging paradigm (Retter and Rossion, 2016; Quek et al., 2018b; Retter et al., 2020). Thus, we summed the baseline-corrected amplitudes of seven consecutive harmonics (i.e., 1.5, 3, 4.5, 6, 7.5, 9, and 10.5 Hz) for the face categorization response, and four consecutive harmonics of 12 Hz up to 48 Hz for the general visual responses of each of the 128 channels. Going forward, the general visual responses and face categorization responses will refer to these aggregated responses. We averaged the responses in the Low-to-High and High-to-Low contrast conditions according to the image contrast value. In other words, responses at step 1 in the Low-to-High condition were averaged with responses at step 14 in the High-to-Low condition, step 2 in Low-to-High with step 13 in High-to-Low, and so forth.

#### Regions of interest

As in previous face categorization frequency-tagging studies (Rossion et al., 2015), the scalp topography of both the general visual and face categorization responses was comparable across observers; still there were moderate individual differences likely because of, e.g., dipole generator orientation, cortical folding, skull thickness, and tissue conductivity (Luck, 2005; Nunez and Srinivasan, 2006; Woodman, 2010; Hauk et al., 2021). We therefore defined unique ROIs for each individual based on their EEG responses in the full-contrast condition. Thus, for each participant and for each type of response (general and categorization), we selected the four EEG channels showing the strongest response amplitude in the full-contrast sequences, and averaged amplitudes across these. ROIs selected this way replicated the previously reported dissociation between the general visual response (12 Hz) and the face categorization response (1.5 Hz; Rossion et al., 2015; Retter and Rossion, 2016; Quek et al., 2018b see Extended data Figures 5-1 and 5-2 for the list the four channel ROIs selected for each individual in the Natural and Negative Contrast group, respectively. Asterisks indicate participants with duplicate channels across the general visual response and face categorisation ROIs.). Overall, ROIs for the general visual response were located over medial occipital channels (the four channels most commonly selected for observers were Oiz-O2-POI2-Iz for natural full-contrast and Oiz-Oz-POI1-O1 for negative full-contrast) whereas ROIs for the face categorization response clustered over bilateral occipito-temporal channels (most commonly selected were channels P10-PO10-PO12-PO9 for natural full-contrast and PO12-P10-PO10-PO11 for negative full-contrast). There was little overlap between the ROI of the general visual response and the categorization response (5.83% overlap between channels in natural contrast group and 3.33% overlap in negative contrast group).

We investigated the hemispheric lateralization of the face categorization response. To ascertain that the potential hemispheric differences result from functional lateralization, and not from differences in channel selection across hemispheres and individuals, we determined symmetrical right and left ROIs based on the group-averaged face categorization responses in the full-contrast condition, i.e., the same channels were averaged for all participants for this analysis. The right occipito-temporal (ROT) ROI was composed of channels PO8, PO10, PO12, and P10, while the left occipito-temporal (LOT) ROI was composed of channels PO7, PO9, PO11, and P9.

#### Definition of minimal and optimal contrast

In each ROI and for each response, we defined the minimum and optimum levels of contrast for each neural response separately. The minimum contrast was the lowest contrast step to elicit a general visual or categorization response amplitude significantly larger than 0 μV. The optimal contrast was defined as the contrast step from which general visual or categorization responses were not significantly different to responses to full-contrast stimuli. Note that full-contrast and individual contrast step amplitude values are based on different numbers of trials (*n* = 56 and *n* = 24, respectively), likely resulting in a difference of SNR. Yet, since this was the case in both the natural and negated samples, any potential SNR difference is expected to be similar across them, and unlikely to contaminate our finding of a relative group difference in the face categorization response dynamics. In Extended Data Figure 5-3, we confirm that the amplitude of the face categorization response in full contrast sequences is robust to subsampling.

The contrast minimum and optimum were determined at the group-level using a bootstrapping procedure. First, for each type of response and in each experiment, we sampled 15 subjects with replacement from the original dataset. Next, we computed the 95% confidence interval of the mean of the bootstrapped sample at each contrast step of the sweep sequence. The contrast minimum was defined as the lowest contrast value where the response’s 95% confidence interval exceeded zero. Similarly, we defined the optimum as the lowest contrast at which the 95% confidence interval included the mean amplitude of the full-contrast response in this bootstrapped sample. We repeated this entire process 10,000 times. Finally, we extracted the peak of the distributions of bootstrapped minimum and optimum values of the general visual or face categorization responses.

#### Response amplitude comparisons

In addition to determining the minimum and optimum contrast, we examined whether the magnitude of the general and categorization responses differs across conditions. We expected the influence of contrast negation on the neural response amplitude to emerge at specific contrast levels, and show some stability across consecutive contrast levels, we ran permutation tests with cluster-based correction on the relevant datasets (for a detailed description of the procedure, see Maris and Oostenveld, 2007). However, since we were aware that the stringent cluster-based correction might favor the null hypothesis, we additionally report noncluster-based statistics.

Briefly, we obtained the distribution of *t* statistics under the null hypothesis of an absence of difference between conditions by permuting the condition labels of observer data 10,000 times. For each permutation iteration, we calculated the two-tailed *t* statistic (for either pairwise or independent sample comparisons, depending the comparison) across all contrast steps, with an α of .05. We then saved the largest summed *t* value among clusters of consecutively significant contrast steps. Finally, we computed the summed *t* values within significant clusters found in the nonpermuted data (α = 0.05) and calculated their respective *p*-values as their percentile relative to the permutation distribution of maximum cluster *t* values.

Additional statistical analyses were conducted using mixed model or repeated measures ANOVAs. The Greenhouse–Geisser correction was applied whenever sphericity was violated and *p*-values were corrected via Bonferroni whenever applicable.

## Results

In a fast periodic presentation paradigm, we measured the implicit neural signatures of the categorization of natural human face images (presented at a rate of 1.5 Hz) in a rapid 12-Hz stream of natural images of objects. We took advantage of periodic brain activity to extract the neural responses elicited by the image stream (i.e., the general visual response) and, most importantly, the periodic deviations from this response by the faces appearing at 1.5 Hz (i.e., the face categorization response; see Fig. 4). We progressively modulated image contrast to estimate the amount of sensory input necessary for each type of neural responses to emerge (minimum) and reach its full-fledged amplitude (optimum) in the present design. Image sequences were presented either in their natural contrast polarity, or in a reversed, negative, contrast polarity. By comparing the minimum and optimum values of contrast for each neural response to emerge and reach their optimum, in natural and negative sequences, we quantified the influence of natural statistics on the dynamics of visual processing when categorizing faces in fast streams of naturalistic, in-the-wild, stimuli.

### Face categorization response in full contrast viewing conditions

**Figure 5.**
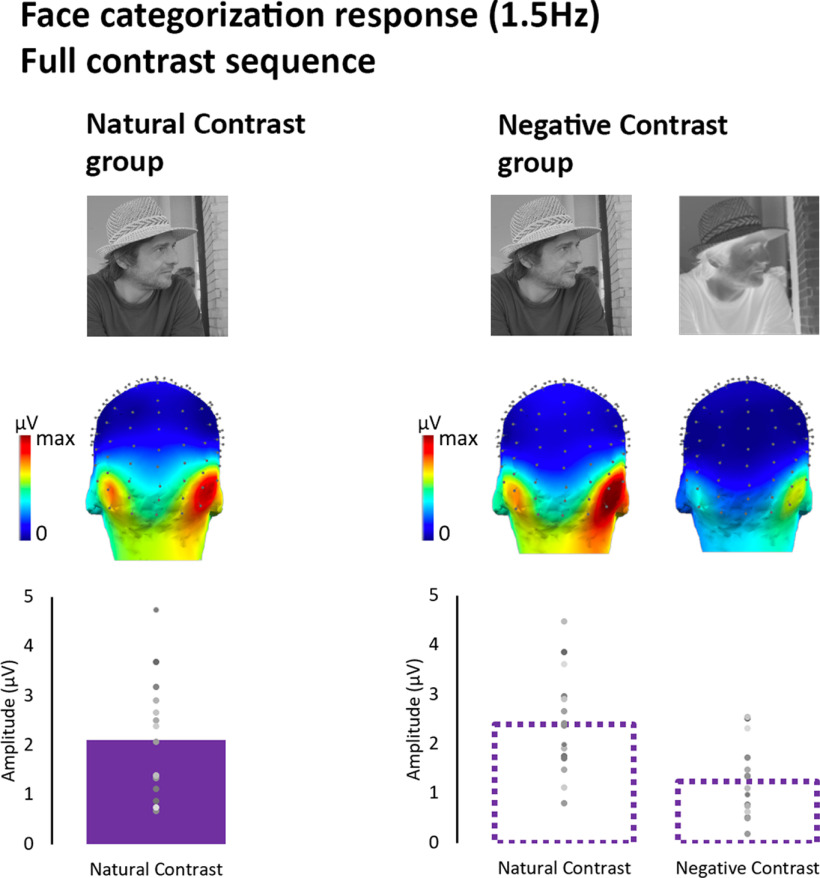
Face categorization response (1.5 Hz) in the full-contrast stimulation sequences, in natural and negative contrast groups. *top,* Group-level averaged scalp topographies. Bottom, Mean baseline-corrected amplitudes averaged within individually defined ROIs (see Materials and Methods). Dots represent individual data points.

The face categorization response reflects the brain’s ability to consistently discriminate face images, despite their diversity, from the other object categories shown in the stimulus sequence (Rossion et al., 2015, 2018). Across experiments, the responses to the full-contrast sequences of naturally-contrasted images were used as references to define the amplitude of the fully-fledged face categorization response, i.e., its amplitude at the maximum level of the tested contrast range. All observers showed periodic responses at the frequency of face presentation and its harmonics; all harmonic frequencies were aggregated to quantify the face-selective EEG response at the individual level (see Materials and Methods; Fig. 4). In line with previous studies (Liu-Shuang et al., 2015a,b; Quek et al., 2018b; Retter et al., 2020; Or et al., 2021; Rekow et al., 2022), the group level categorization response was most prominent over the bilateral occipito-temporal scalp electrodes (Fig. 5). We defined occipito-temporal ROIs for each individual based on their response to full-contrast sequences.

10.1523/ENEURO.0420-21.2022.f5-1 Extended Data Figure 5-1 Individual ROIs in the Natural Contrast group. * = Participants which duplicate channels across general visual response ROIs and face categorisation ROIs. Download Figure 5-1, DOCX file.

10.1523/ENEURO.0420-21.2022.f5-2 Extended Data Figure 5-2 Individual ROIs in the Negative Contrast group. * = Participants which duplicate channels across general visual response ROIs and face categorisation ROIs. Download Figure 5-2, DOCX file.

10.1523/ENEURO.0420-21.2022.f5-3 Extended Data Figure 5-3 Halving the number of full-contrast trials does not influence the amplitude of the face categorization response. The mean amplitude of the face categorization response to full-contrast stimulation sequences was stable whether based on all trials or only (odd/even) half of them, in natural and negative contrast groups (in individually defined ROIs; see Methods). Dots represent individual response amplitude values. The number of full contrast and sweep trials (*n* = 56 and *n* = 24, respectively) differed equally across groups; any potential SNR difference arising due to this difference of trial number should thus be similar across groups, and not contribute to our main findings. Nevertheless, we subsampled trials from the full-contrast condition in order to investigate whether the amplitude of the face categorization response was influenced by subsampling. To do so, we split full contrast trials into odd and even 'steps' and tested whether face categorization response amplitude was similar across sampling methods (odd, even, all trials). As shown in the figure below, face categorization response in full contrast sequences is relatively stable in amplitude irrespective of the number of trials. In a mixed ANOVA with group (natural contrast, negated contrast) and sampling method (odd, even, all) as factors, we confirmed that the sampling method did not influence the amplitude of the face categorization response significantly (*F*_(1.37,38.39)_ = 2.69, *p* = .1, η^2^ = .09). Furthermore, the interaction between sampling method and group was non-significant (*F*_(1.37,38.39)_ = .83, *p* = .4, η^2^ = .029). While null results should always be taken with caution, they suggest that the face categorization response we measured in full contrast trials across groups was stable whether the full sample of trials was used or if we halve it to get closer to the n of sweep trials. Download Figure 5-3, TIF file.

Although there was a slightly larger amplitude overall in the negative contrast group for the full natural contrast sequences (Fig. 5), a one way ANOVA with Group (natural group, negative group) as between-subject factor did not reveal any difference across natural and negative contrast groups (*F*_(1,28)_ = 0.522, *p* = 0.476, η^2^_p_ = 0.018).

Furthermore, since the participants in the negative contrast experiment viewed a full-contrast sequence with both natural images and their contrast-negated versions, we were able to examine the impact of contrast negation on the full-contrast face categorization response. A one-way repeated-measures ANOVA with Contrast polarity (natural vs negated) as within-subject factor revealed a significant main effect of Contrast polarity (*F*_(1,14)_ = 26.53, *p* < 0.0001, η^2^_p_ = 0.65) with contrast negation reducing the amplitude of the face categorization response by 47% (±13%) on average (see Fig. 5).

These findings confirm the drastic effect of contrast negation on face categorization, quantifying it for the first time here with natural images. It further demonstrates that at least half of the amplitude of the face categorization response is not accounted for merely by the physical parameters of the stimulus but reflects the successful match between the incoming input and the stored representation of what a face generally looks like.

### Contrast negation influences both the minimal and optimal contrast for human face categorization

**Figure 6.**
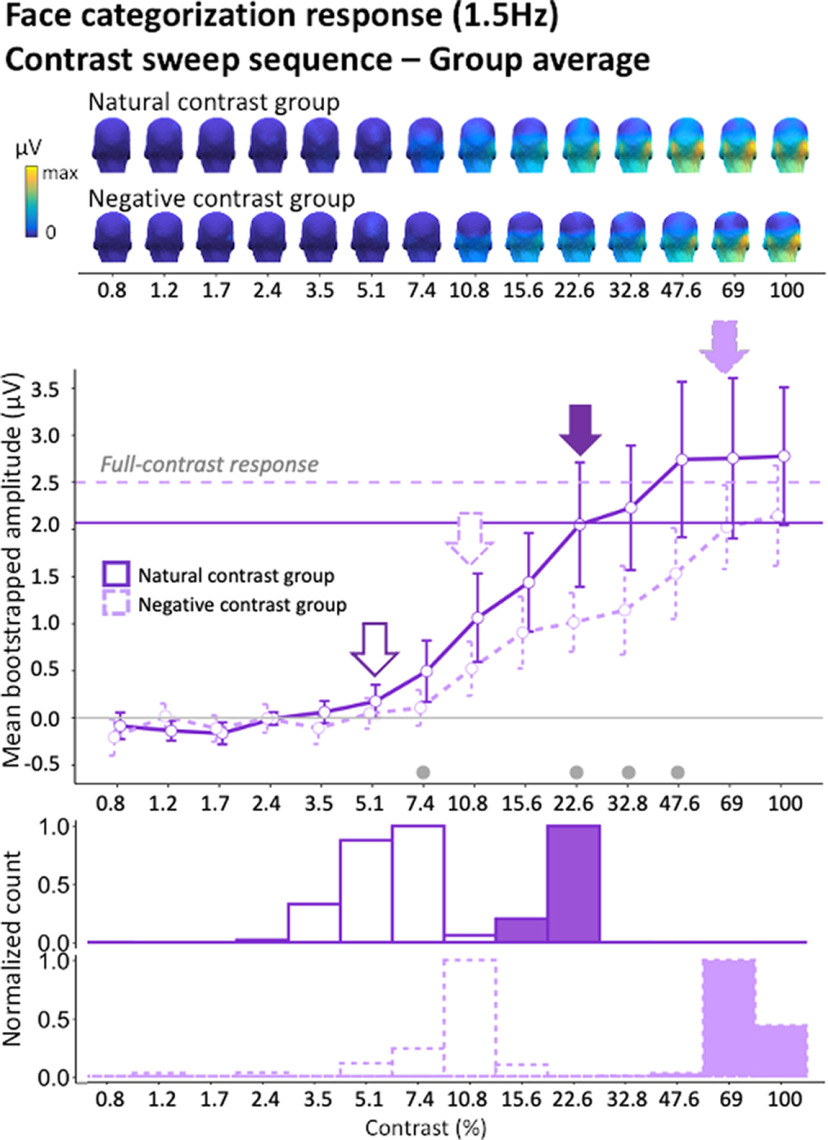
Group-level face categorization response in contrast sweep sequences in natural and negative contrast groups. Top, Scalp topographies at different contrast steps. Bottom, Line plots depict the profile of the bootstrapped mean face categorization response (averaged within individually defined ROIs; see Materials and Methods) as a function of contrast (plain line for natural contrast and dotted line for negative contrast). Open and filled arrows indicate the minimum and optimum levels of contrast, respectively. Error bars represent mean bootstrapped 95% confidence intervals. Dots below the response curves represent significant amplitude differences between natural and negative contrast group responses at each contrast steps (gray dots for significance at *p* < 0.05 with no cluster-based correction; see Materials and Methods). Bar graphs represent the peak of the bootstrapped distributions (normalized within a range of 0–11) of the contrast minimum (open bars) and optimum (filled bars) in natural and negative contrast groups. Face categorization based on negative contrast images elicited weaker response amplitudes and raised minimum and optimum thresholds relative to natural contrast images.

**Figure 7.**
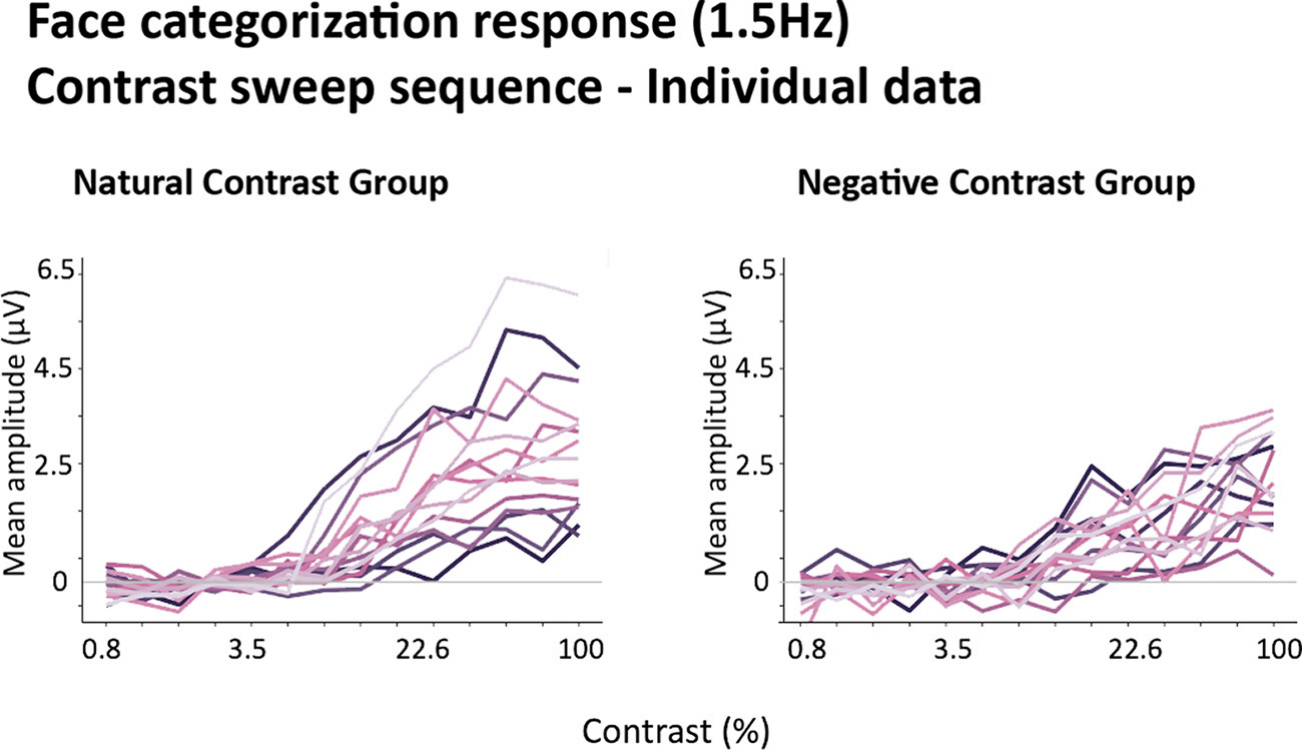
Line plots of the individual face categorization responses (1.5 Hz) in contrast sweep sequences in natural and negative contrast groups.

The profile of the 1.5-Hz face categorization response in the contrast sweep sequences is plotted separately for the natural and negative contrast polarity groups (Fig. 6). In the natural contrast experiment, face categorization responses hovered near zero in the lower end of contrast continuum and became significant already at 5.1% of contrast. This response increased with further image visibility until it no longer differed from full-contrast face categorization responses at 22.6%. Thus, under ecological contrast polarity, the categorization of faces required a minimum contrast of 5.1% to emerge and reached full-contrast levels at only 22.6% of contrast. In comparison, as illustrated in Figure 6, the face categorization responses to contrast-negated images required not only a higher contrast to emerge (10.8%) but a much higher level of contrast (69%) to reach full-contrast response levels. These findings suggest that the disruption of natural statistics did not only increase the amount of minimal sensory input that is necessary to elicit a categorization response but also drastically protracts the dependence of the categorization response on contrast, suggesting a less efficient visual process. While there was some degree of interindividual variability in the amplitude and steepness of EEG response as a function of contrast, the response profiles were largely consistent across participants (Fig. 7).

The observation that the categorization of contrast-negated face images elicits neural responses with an amplitude that, at high contrast (69%; 100%) almost reaches the amplitude of the categorization response to natural images may seem surprising. Since contrast negation decreased the face categorization response by half in full-contrast sequences, one could have expected the categorization responses to contrast-negated in sweep sequences to never reach the categorization response amplitude for natural images in full contrast sequences. Yet, contrast negation reduced the amplitude of the face categorization response in the contrast sweep sequences even at most supra-threshold contrast levels, showing again the detrimental effect of contrast negation on categorization in sweep sequences (Fig. 6; uncorrected *p*-value range = 0.007–0.047; these differences just failed to reach the cluster-wise significance minimum: cluster *p*-value range = 0.06–0.3).

### Hemispheric lateralization of the face categorization response

**Figure 8.**
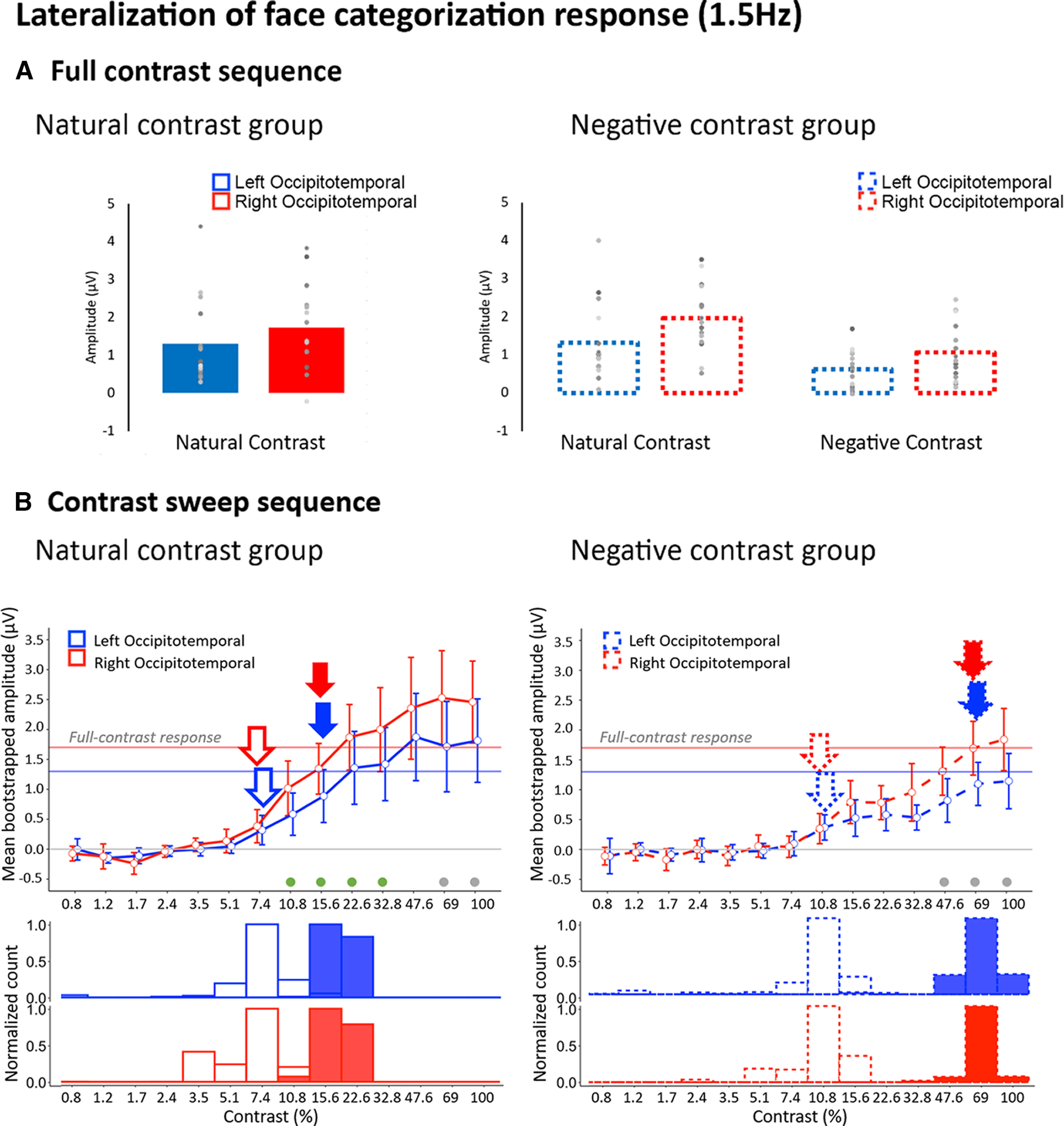
Group-level face categorization responses (1.5 Hz) for LOT (in blue) and ROT (in red) ROIs, defined at the group-level (see Materials and Methods). ***A***, Full contrast sequences. Group-averaged amplitudes in LOT and ROT ROIs in the natural contrast group (left panel) and negative contrast group (right panel). Dots represent individual response amplitude values. ***B***, Contrast sweep sequence. Left panel, Natural contrast group. Bootstrapped sweep response profiles and corresponding minimum distributions for LOT and ROT group-level ROIs. The mean bootstrapped responses in the LOT and ROT are plotted in the top row (error bars represent mean bootstrapped 95% confidence intervals). Open and filled arrows indicate the minimum and optimum levels of contrast, respectively. The dots below the response curves represent significant hemispheric differences in amplitude (gray dots for significance at *p* < 0.05; green dots for *p* < 0.05 significance following cluster-based correction; see Materials and Methods). The rows below represent the bootstrapped distributions of the contrast minimum (open bars) and optimum points (filled bars) in the LOT (middle row) and the ROT (bottom row). Note that the histograms have been separately normalized within a range of 0–1. Right panel, Negative contrast group. Bootstrapped sweep response profiles and corresponding minimum distributions for LOT and ROT ROIs, displayed using the same conventions as in ***A***.

To address the potential hemispheric differences in the modulation of the face categorization as a function of contrast, we computed the minimum and optimum contrast levels within LOT and ROT ROIs in natural and negative contrast experiments, separately (Fig. 8).

These analyses were conducted on symmetric ROIs predefined at the group level (see Materials and Methods) to ensure that hemispheric differences in face categorization responses reflect genuine functional lateralization, and not individual differences in channel selection. The resulting group-level ROIs contained channels with weaker amplitudes than the individual ROIs, since the latter were selected to contain the largest response amplitudes at the individual level.

First, we tested the hemispheric lateralization of the face categorization response to natural full-contrast sequences (Fig. 8*A*). To do so, we conducted a mixed model ANOVA with Hemisphere as a within-subject factor and Group (natural group, negative group) as a between-subject factor. We found a significant main effect of Hemisphere (*F*_(1,28)_ = 7.14, *p* = 0.012, η^2^_p_ = 0.2), with larger amplitudes over the right than the left hemisphere. Overall the face categorization response in natural full-contrast sequences was of similar magnitude across groups, corroborating the findings with the full-contrast responses in individual ROIs (*F*_(1,28)_ = 0.13, *p* = 0.72, η^2^_p_ = 0.005). The interaction between Hemisphere and Group was not significant (*F*_(1,28)_ = 0.26, *p* = 0.61, η^2^_p_ = 0.009).

We additionally tested the effect of Contrast Polarity as a within-subject factor in the full-contrast data of the negative contrast group. The 2 × 2 repeated measures ANOVA with Contrast polarity (natural vs negative contrast experiment) and Hemisphere (LOT vs ROT) as within-subject factors revealed a significant main effect of Hemisphere (*F*_(1,14)_ = 25.28, *p* < 0.0001, η^2^_p_ = 0.64) and of Contrast Polarity (*F*_(1,14)_ = 5.8, *p* = 0.031, η^2^_p_ = 0.29). The amplitude of the face categorization response was again larger in the right than the left hemisphere. As expected, full-contrast images presented in their natural contrast polarity resulted in the strongest face categorization response overall (Fig. 8*A*). The absence of interaction between Contrast polarity and Hemisphere (*F*_(1,14)_ = 1.15, *p* = 0.3, η^2^_p_ = 0.08) suggests a comparable influence of Contrast polarity across hemispheres during the viewing of full contrast periodic sequences.

For contrast sweep sequences presented in a natural polarity, our bootstrapping procedure showed that face categorization responses emerged at 7.4% contrast and saturated at 15.6% in both the left and right hemispheres (Fig. 8*B*). The right hemisphere showed higher amplitudes at most contrast levels (from 10.8% to 32.8% of contrast: cluster *p*-value < 0.034). In other words, besides an overall right-lateralization of the face categorization responses, visual processing dynamics when categorizing ecological faces were comparable across hemispheres, at least with the resolution of the logarithmic contrast steps used in the current paradigm.

For contrast-negated image sequences, the face categorization responses emerged later, at a contrast value of 10.8% and reached optimum later, at 69% in both the ROT and LOT regions. Hemispheric differences in amplitude were only significant at the higher ends of the contrast continuum and at an uncorrected *p* value (uncorrected *p*-value range = 0.019–0.04; no clusters reached cluster-based significance minimum, cluster *p*-value = 0.28). Thus, the right hemispheric lateralization of the face categorization response is less reliable overall for negative than natural contrast images.

In sum, we found no consistent impact of contrast negation on the right hemispheric lateralization of the face categorization response. However, it is important to keep in mind that these findings stem from group-level ROIs, which only partially overlap with the maximally-responsive channels as defined at the individual level. Therefore, we cannot exclude the possibility that hemispheric differences would emerge if the maximally responsive channels would perfectly overlap across individuals. Besides addressing the lateralization of contrast negation effects on face categorization, the present results confirm that the disruption of natural image statistics by means of contrast negation protracts the contrast dependence of the neural categorization response. This suggests that the impact of natural contrast statistics on visual processing dynamics replicates regardless of the method used to select scalp electrodes (individual-level vs group-level) and attests the robustness of the findings.

To summarize, the disruption of image natural statistics through contrast negation not only decreased the neural signatures of face categorization at full contrast by almost half of their amplitude, the novel finding is that it influenced the underlying visual processing dynamics by protracting both the emergence (i.e., contrast minimum) and the optimum of the electrophysiological face categorization response to higher contrast levels. Contrast negation drastically expanded the dependence of the electrophysiological categorization response on contrast, and reduced its amplitude at most contrast steps.

### Contrast negation does not affect the 12-Hz general visual response to full-contrast sequences

**Figure 9.**
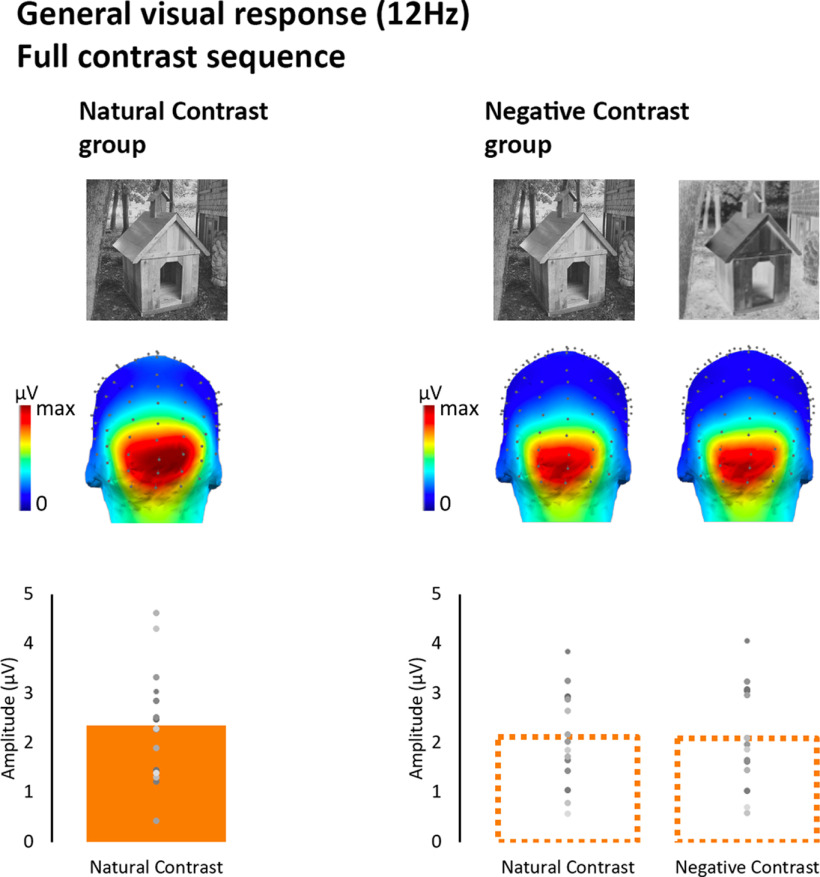
General visual response to the 12-Hz periodic rate in full-contrast stimulation sequences in natural and negative contrast groups. Top, Group-level averaged scalp topographies. Bottom, Mean amplitudes averaged within individually defined ROIs (see Materials and Methods). Dots represent individual response amplitude values.

Besides the face categorization response, all observers showed a neural response at the 12 Hz frequency and harmonics, in response to the 12-Hz image stream (Fig. 4). This neural signal reflects the general response of the visual system to the stimulus sequence, regardless of the category membership of the presented images. As shown in Figure 9, the general visual response to full-contrast images was centered over medial occipital channels.

The medial occipital location of this response suggests that it reflects the low-level encoding of image basic properties such as amplitude spectrum and contours (Ales et al., 2012; Norcia et al., 2015; Hauk et al., 2021), which are comparable between positive and negative polarity images. There was no difference between groups for the 12 Hz neural response to the natural contrast polarity images (*F*_(1,28)_ = 0.38, *p* = 0.54, η^2^_p_ = 0.014). Similarly, in the negative contrast group, there was no effect of Contrast polarity on the 12-Hz general visual response (*F*_(1,14)_ = 0.135, *p* = 0.72, η^2^_p_ = 0.01).

To further establish that contrast negation selectively affected face categorization while leaving generic visual response unchanged in full contrast viewing conditions, we ran a 2 × 2 repeated measures ANOVA with Contrast polarity (natural vs negated) and Response (general visual vs face categorization) as within-subject factors. The Contrast polarity × Response interaction was significant (*F*_(1,14)_ = 30.54, *p* < 0.001, η^2^_p_ = 0.69) confirming that contrast negation selectively reduced the amplitude of the 1.5-Hz face categorization response (P_bonf_ < 0.001), leaving the general visual response to the 12-Hz sequence unaffected (P_bonf_ = 1). In interpreting this interaction, it is important to recall the difference in SNR between the response types (Extended Data Fig. 5-3), which reflects the generally higher number of events contributing to 12-Hz responses compared with 1.5-Hz responses. However, it is unlikely that the interaction reflects the difference in SNR as the effect of negation was only significantly observed for the 1.5 Hz categorization, i.e., the response with the lowest SNR. Main effects were of no interest in the present context; they are only reported here for the sake of completeness (main effect of Response: *F*_(1,14)_ = 0.68, *p* = 0.423, η^2^_p_ = 0.046; main effect of Contrast polarity: *F*_(1,14)_ = 21.58, *p* < 0.001, η^2^_p_ = 0.61).

These findings confirm the selective impact of contrast negation on the face categorization response and thus validates this image manipulation as a means to disentangle the relative contributions of low-level versus high-level visual mechanisms to the presently observed contrast sensitivity profiles.

### Contrast negation increases the minimum level of contrast necessary for the emergence of general visual responses

**Figure 10.**
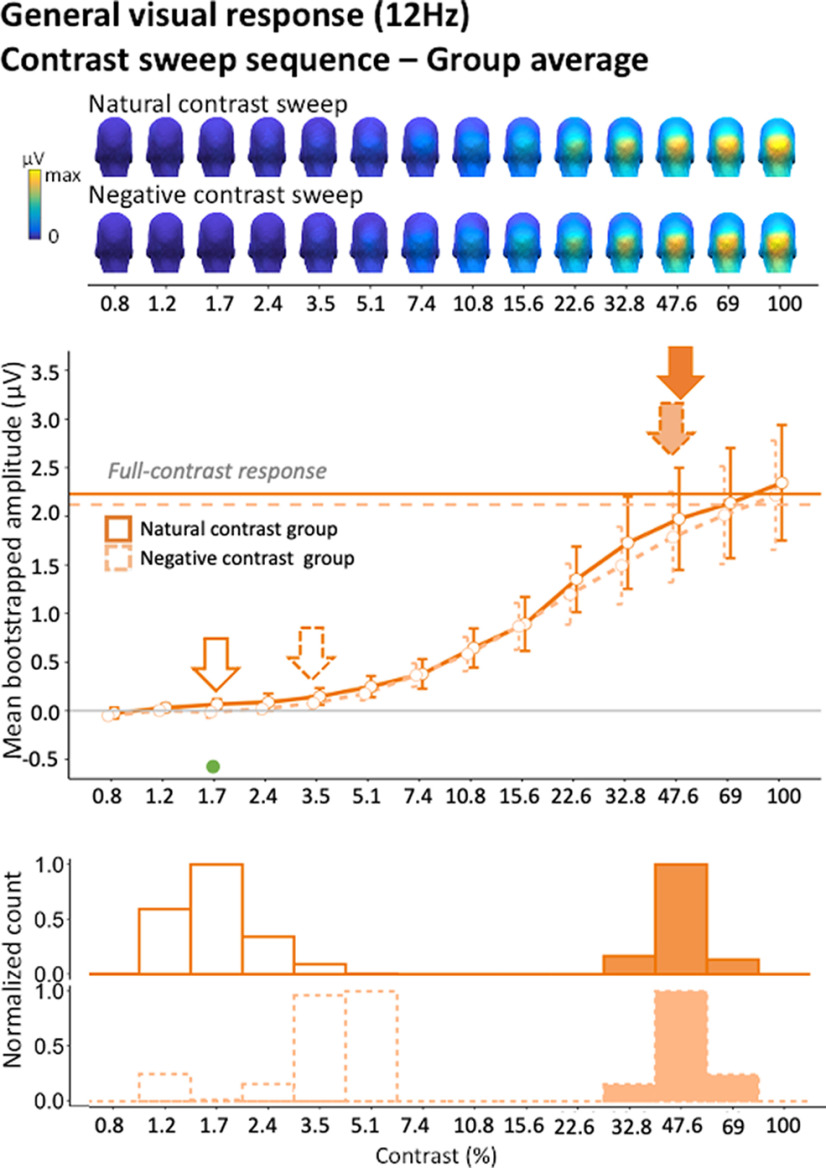
Group-level common visual response (12 Hz) in contrast sweep sequences in natural and negative contrast groups. Scalp topographies at different contrast steps. Line plots depict the profile of the bootstrapped mean face categorization response as a function of contrast (averaged within individually defined ROIs; plain line for natural contrast and dotted line for negative contrast). Open and filled arrows indicate the minimum and optimum levels of contrast, respectively. Error bars represent mean bootstrapped 95% confidence intervals. Dots below the response curves represent significant amplitude differences between natural and negative contrast group responses at each contrast steps (gray dots for significance at *p* < 0.05; green dots for *p* < 0.05 significance following cluster-based correction; see Materials and Methods). Bar graphs represent the peak of the bootstrapped distributions of the contrast minimum (open bars) and optimum (filled bars) in natural and negative contrast groups. Note that the histograms have been separately normalized within a range of 0–11.

**Figure 11.**
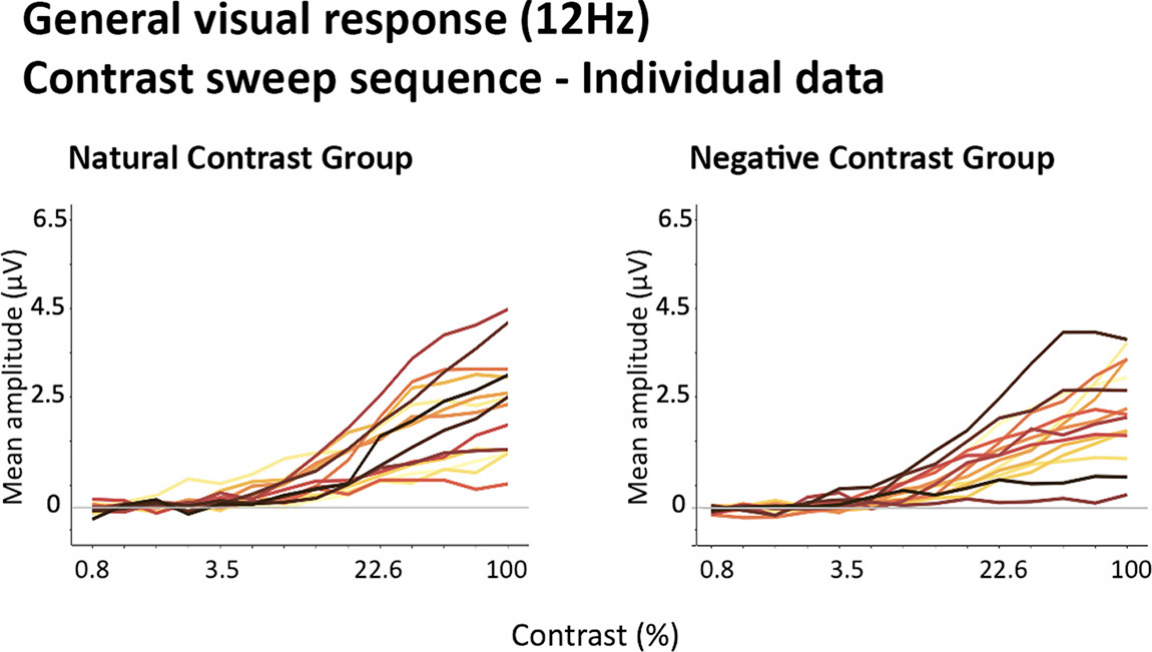
Line plots of the individual common visual response (12 Hz; averaged within individually defined ROIs) profiles in contrast sweep sequences, in natural and negative contrast groups.

Next, we examined how the 12-Hz general visual response evolved as a function of contrast. The response profile of the contrast sweep sequences is plotted separately for the Natural and Negative Contrast polarity groups (Fig. 10). While there was some degree of interindividual variability in the amplitude and steepness of EEG response as a function of contrast, the response profiles were largely consistent across participants (Fig. 11).

In natural image sequences, the group-level general response emerged significantly above zero at 1.7% contrast and then increased steadily until reaching the optimum level of full-contrast responses at 47.6% contrast (Fig. 10). Hence, the general visual processes triggered by the 12-Hz stimulation sequence exhibited sensitivity to a wide range of contrast levels.

When contrast polarity was negated, the general visual response significantly emerged at a higher level of contrast (3.5% of contrast; Fig. 10). Despite this disparity in response onset, the general visual response in the negative contrast experiment rose to optimum at the same contrast level (47.6%) for natural and negative images. In terms of response amplitude, only a single contrast step differed significantly between experiments (step 3 = 1.7% contrast, cluster *p*-value < 0.046).

## Discussion

The present study reports a systematic investigation of the influence of natural image statistics on the visual processing dynamics occurring during human face categorization. Reversing the natural polarity of image contrast severely disrupts face categorization and reduces brain responses to faces compared with when their natural contrast polarity is preserved. Yet, how natural contrast statistics contribute to visual processing during naturalistic categorization has been so far elusive because of two main reasons. First, the effect of contrast negation on face categorization has typically been investigated using restricted sets of segmented images of faces and objects; these testing conditions are very different from everyday life, where faces and objects have to be categorized in a cluttered environment and despite a wide variety of appearances. A second limitation is that past studies did not investigate the nature of the facilitation triggered by natural statistics. It is unclear whether knowledge of natural statistics decreases the amount of sensory input that is necessary to see the face categorization emerge in the observer’s brain (i.e., earlier onset), if it reduces the sensory input needed to generate a fully-fledged categorization response (i.e., earlier optimum), or both, making the categorization less dependent on contrast overall. We found that natural contrast statistics cause a drastic reduction in the amount of sensory input required for face categorization responses to emerge and also reach optimum, therefore severely reducing the minimum/optimum distance.

The medial occipital neural response elicited by the 12-Hz image stream and thought to originate primarily in low-level visual cortex (Hauk et al., 2021), increased monotonically with stimulus contrast in both natural and negated sequences. Contrast negation induced a moderate shift in the onset of the general visual response toward higher contrast values, but did not affect the level of contrast required to elicit an optimal general visual response or its overall magnitude. It is unclear whether this shift in the emergence of the general visual response is because of a differential activation of the center-surround receptive fields in the retina or the lateral geniculate nuclei (Meister and Berry, 1999), and/or whether it is because of contrast negation hindering general, low-level visual processing in the cortex (e.g., perception of brightness, Yang and Purves, 2004; Pickard-Jones et al., 2020; orientation, Girshick et al., 2011; apparent contrast, Bex and Makous, 2002; Haun and Peli, 2013). Hence, shading, i.e., the pattern of luminance across a surface, is informative of the structural properties of many things around us, not just faces. Combined with the prior assumption that light comes from above in the natural environment, the human visual system forges strong priors of how shape should be interpolated from shading in everyday life. For example, contrast negation impedes texture perception (Balas, 2012), and local shape computations based on texture cues (Fleming et al., 2004). Shading disruption through negation is therefore expected to disrupt the general visual processes reflected by the medial occipital 12-Hz response at least to some extent, especially when the stimuli are diverse, cluttered and complex scenes, i.e., challenging viewing conditions that maximally tap into prior experience (Hupé et al., 1998; Wyatte et al., 2014).

The impact of contrast negation was much more spectacular for human face categorization responses. For naturally-contrasted images, the face categorization response emerged at 5.1% of contrast (RMS contrast of 0.009) and quickly reached its optimal amplitude (22.6% or 0.041 RMS contrast). Considering the complexity and wide variety of face images in the sequence, these values are intriguingly low. It indicates that when the sensory input matches ecological regularities, the human brain is able to categorize, i.e., to consistently differentiate highly variable faces from other visual categories, even in seemingly suboptimal, low-contrast, viewing conditions.

In natural contrast viewing conditions, the rise in face categorization response was fast but gradual as it only reached its fully-fledged amplitude four steps later, at 22.6% of contrast. This profile indicates the progressive but rapid direction of categorization by natural statistics. Past evidence indicates that a fast rise in the face categorization response amplitude results from a proportionately rapid increase in the number of faces that are correctly categorized in the rapid image sequences (Retter et al., 2020). Whether such rise of the face categorization response reflects an increase in the sensitivity to face images, in the tolerance to face image variability, or both cannot be teased apart in the present study.

Our findings also imply that there is at least a restricted range where face categorization is contrast-dependent. This disagrees with previous evidence suggesting that the high-level cortical regions involved in the categorization of complex stimuli are (quasi-)invariant to basic properties of the stimulus such as, e.g., contrast, position, size to enable efficient categorization (Ito et al., 1995; Andrews and Ewbank, 2004; Kovács et al., 2008). Instead, our findings aligns with several studies that showed that high-level stages of visual processing are at least to some extent sensitive to contrast (Rolls and Baylis, 1986; Avidan et al., 2002; Murray and He, 2006; Yue et al., 2013), and with the more nuanced stance suggested by Näsänen et al. (2006) that human face identification is influenced by contrast only at low contrast levels (RMS < 0.1). Here, the RMS value at which the face categorization response reached its optimum level was 0.041, thus at a lower level than the one reported by Näsänen et al. (2006). This difference is most probably explained by methodological aspects. Näsänen and colleagues (2006) explicitly instructed their participants to recognize the identity of faces flashed in homogeneous sequences of segmented face images. The basic level of categorization, the high stimulus heterogeneity, as well the reliance on implicit neural measures likely explain the lower cutoff RMS value found here. Leaving aside these aspects, our finding that the face categorization response did not reach its optimum as soon as it emerged (minimum) is in line with the conclusions of these authors, namely that the underlying mechanisms are contrast-variant at the lower ends of the contrast continuum (RMS < 0.041), and resistant to contrast at higher levels of contrast.

In agreement with previous evidence, we found that the disruption of natural statistics because of contrast negation reduced by about a factor of 2 the overall amplitude of the face categorization response (Liu-Shuang et al., 2015a). More interestingly, contrast negation doubled the minimum level of contrast necessary for the human brain to categorize a face as a face. While face categorization responses emerged at 5.1% of contrast (RMS of 0.009) in natural contrast settings, a minimum of 10.8% (RMS of 0.019) of contrast was required in negated streams. Furthermore, contrast negation expanded visual processing, shifting the optimum of the categorization response to 69% (RMS of 0.124), which is three times the optimal contrast for face categorization in natural contrast settings (22.6%; RMS of 0.041).

Altogether this indicates that the access to internally stored knowledge about how faces appear in real-life, i.e., their natural statistics, does not merely enable stronger output responses, but actually empowers a faster and more efficient visual processing for the purpose of categorization. When these natural statistical priorities fail to guide visual processing, much more sensory input (contrast) is needed, which weakens and pushes the neural categorization response to a considerably higher contrast. In contrast to its dramatic effect on the dynamics of the face categorization response, we found no consistent impact of contrast negation on right hemispheric specialization in face processing. This null finding must be interpreted with caution but suggests that the right-hemispheric specialization of face processing is not triggered by the contrast ordinal relationships of the human face.

In the fast periodic visual stimulation paradigm used here, each image appears very briefly and is masked both by the preceding and succeeding images (similar to rapid serial visual presentation; Potter and Levy, 1969; Keysers et al., 2001, 2005; Potter et al., 2014). This implies that most of the facilitation observed for the categorization of natural images happened during the feedforward transmission of the visual signals (Lamme and Roelfsema, 2000; Keysers and Perrett, 2002; Bacon-Macé et al., 2005; Fahrenfort et al., 2007; although for monkey electrophysiological evidence of rapid feedback, see Girard et al., 2001; Hupé et al., 2001). Several authors have discussed the possibility of the influence of natural statistics priors in a purely feedforward architecture, with each ventral stream stage amplifying the features (and contingencies of those) that are relevant for categorization (Simoncelli and Olshausen, 2001; Martin et al., 2018; but see Kar et al., 2019). Clarifying the exact mechanisms of such amplification is out of the scope of the present work. Yet, the fact that natural statistics influenced the dynamic visual processing leading up to categorization but seemed to only moderately guide the general visual responses indicate that most of such amplification occurs when signals are read out by the high-level visual cortex responsible for categorization.

As human observers experience that members of a visual category share similar shape and function, access to such natural statistics is likely to facilitate visual processing dynamics for their categorization. Indeed, whether an input corresponds to stored natural statistics determines its categorizability. Since the current study was limited to categorizing human faces, our findings may not apply to other (visual) categories. However, we speculate that the facilitation of processing dynamics for other visual categories is unlikely to be as clear as for faces. Indeed, face categorization is one of the most critical visual functions for human social adaptation, and this likely exerted a strong evolutionary pressure for the development of fast and efficient categorization mechanisms. This may explain why the human species has developed an exceptional experience with and sensitivity to the universal properties of face stimuli as a strict and ordered alternation of contrast (Fig. 1; Watt, 1994; Dakin and Watt, 2009; Gilad et al., 2009). Figure 1 illustrates the universality of this property and suggests that the current results can be generalized to all faces, regardless of skin tone. Such priors strongly drive the human visual system as illustrated by face pareidolia (Rekow et al., 2021). Nonface visual categories do not seem to lend such strict contrast rules, i.e., their processing is far less affected by contrast negation (i.e., dogs, Robbins and McKone, 2007; chairs, Itier et al., 2006), artificial objects (i.e., “Greebles”; Vuong et al., 2005), or abstract visual objects (i.e., “blobs”; Nederhouser et al., 2007; Yue et al., 2013). This implies that the influence of contrast negation observed here on the electrophysiological markers of face categorization are mostly driven by the face images. Nevertheless, nonface categories may share other visual regularities, stored in the human visual system as their natural statistics (see Long et al., 2018 for evidence that mid-level curvature features driving categorization into (in)animate categories). However, since the current study did not systematically compare the impact of natural contrast statistics for the categorization of face and nonface categories, whether such natural statistics facilitate visual processing as vigorously as for face stimuli requires further investigation.

How do natural statistics contribute to the visual processing dynamics leading up to human face categorization? Here, we show that natural statistics not only boost the amplitude of face categorization responses in the human brain, but also modulate the underlying processing dynamics by halving the minimal amount of sensory input required to categorize faces. Once this minimum has been reached, the face categorization response samples further input, but reaches its optimum rapidly, i.e., at a three times lower level of contrast than for contrast-negative faces. These results have important implications for how internally stored natural statistics facilitate visual processing for rapid and efficient categorization.

## Synthesis

Reviewing Editor: Satu Palva, Helsingin yliopisto Decisions are customarily a result of the Reviewing Editor and the peer reviewers coming together and discussing their recommendations until a consensus is reached. When revisions are invited, a fact-based synthesis statement explaining their decision and outlining what is needed to prepare a revision will be listed below. The following reviewer(s) agreed to reveal their identity: Mate Gyurkovics.

The manuscript addresses the contribution of natural contrast statistics to face categorization using 12 Hz streams of natural nonface image and periodically inserted examplars of faces at 1.5 Hz. The study is well designed, the analyses justified and robust, and the claim cautious and corroborated by their findings and existing literature. The authors have made a thorough revision of the manuscript based on previous comments. Especially appreciated are the additional analyses that were run to address some exploratory notions. However, there are some core issues that were not addressed fully that should still be considered.

The paragraph starting line 649 could gain some clarity. The authors idea in these lines (the linear increase of the face-categorization response is not “all-or none” but the response to each face might be, following the ref paper) is comprehensible, but the way it is described is not very straighforward (maybe the description of the face-categorization response could be described using an other term than “all-or-none” to avoid ambiguity). Most importantly, the authors should provide a clearer explanation in the introduction of why they believe their methodology captures the dynamics of evidence accumulation and what they mean by evidence accumulation in general. They should clearly discuss and separate single-trial face categorization (which may or may not be all-or-none) vs. the neural response to the face sequences across different contrast steps (which may or may not be all-or-none as well), but also why they would consider the latter a measure of evidence accumulation. A change in the dynamics of evidence accumulation would suggest a change in the rate of the evidence accumulation process (drift rate in drift-diffusion modelling terms) across conditions. However, the present study provides no evidence for this, nor does it set out to do this, given that the authors claim evidence accumulation rate is fixed by stimulus presentation rate in their task (which may or may not be the case, as evidence accumulation may be faster than presentation rate or may continue after offset, although these are both admittedly unlikely). Nevertheless, the authors do seem to imply on line 693 that there is faster evidence accumulation in the natural contrast than in the contrast negated condition, which may not be justified. In this same line, evidence accumulation is also described as more efficient in the natural contrast condition - but it is left unclear what exactly this means. Isn’t it, in fact, just the quality and quantity of the available evidence that differs between the two conditions (leading to fewer correct categorizations in one case compared to the other), rather than the process itself of how that evidence is extracted and used? Or do the authors consider these two things equivalent?

In the same vain, in lines 694-695, the authors say that “much more sensory evidence (contrast) must be accumulated” when natural statistics are corrupted, and this interpretation seems to be in line that it is not the nature/speed/dynamics of evidence accumulation that changes, simply the amount of evidence that is available (or maybe the decision criterion, etc.). Relatedly, it is also a little confusing to refer to “contrast” being accumulated since contrast is a fixed characteristic of each image.

Notably, early on in the paper, the authors introduce their methodology as capturing two key aspects of face categorization, aggregated over multiple stimuli: sensitivity to faces relative to other categories, and tolerance to high variability in face appearance (lines 116-118). It would be helpful to interpret their findings in these terms, e.g., sensitivity to face stimuli might decline in the contrast negated situation compared to natural contrast, or tolerance to variability may decrease (e.g., more canonical face representations are preferred when contrast information is unusual), or both (unfortunately, these cannot be teased apart, as there is no information on individual face categorizations). They may also link these terms explicitly to aspects of evidence accumulation, which I think might clear up some of my confusion.

Relatedly, it is still somewhat unclear to me why the authors do not consider the alternatives introduced on lines 142-146 to be competing, mutually exclusive ideas. One posits an all-or-none type of face response, the other a face response that keeps increasing even after a categorization response has been reached.

One other issue that may be addressed in more detail is the domain-specificity of findings. While authors provide data supporting the idea that natural contrast statistics help face categorization while having little impact on a general visual response, and the authors do indeed acknowledge that this could be the case for other categories as well, it would be important to explicitly mention that this study does not provide evidence for the - otherwise well established - prioritized (or unique) nature of face processing, given that face processing was not contrasted against another category. As hinted at the end of the paragraph starting on line 718, future studies are required to examine whether face categorization is more sensitive to natural contrast statistics than the identification of other categories (e.g., buildings, animals, plants).

In the results section, authors have added additional comments on lines 575-580, however, it should still be made explicit there that the interpretation of the interaction is hampered by the fact that the two levels of Response likely have different SNR. Just as a reminder to readers.

Finally, a minor thing but in Fig. 3A, the unit of time is indicated to be ms, but the figure appears to show seconds.
