# Supplementary figures and images for: Natural Contrast Statistics Facilitate Human Face Categorization
Source: eNeuro. 2022 Oct 4;9(5):ENEURO.0420-21.2022. doi: 10.1523/ENEURO.0420-21.2022 (PMC9536856; doi:10.1523/ENEURO.0420-21.2022)

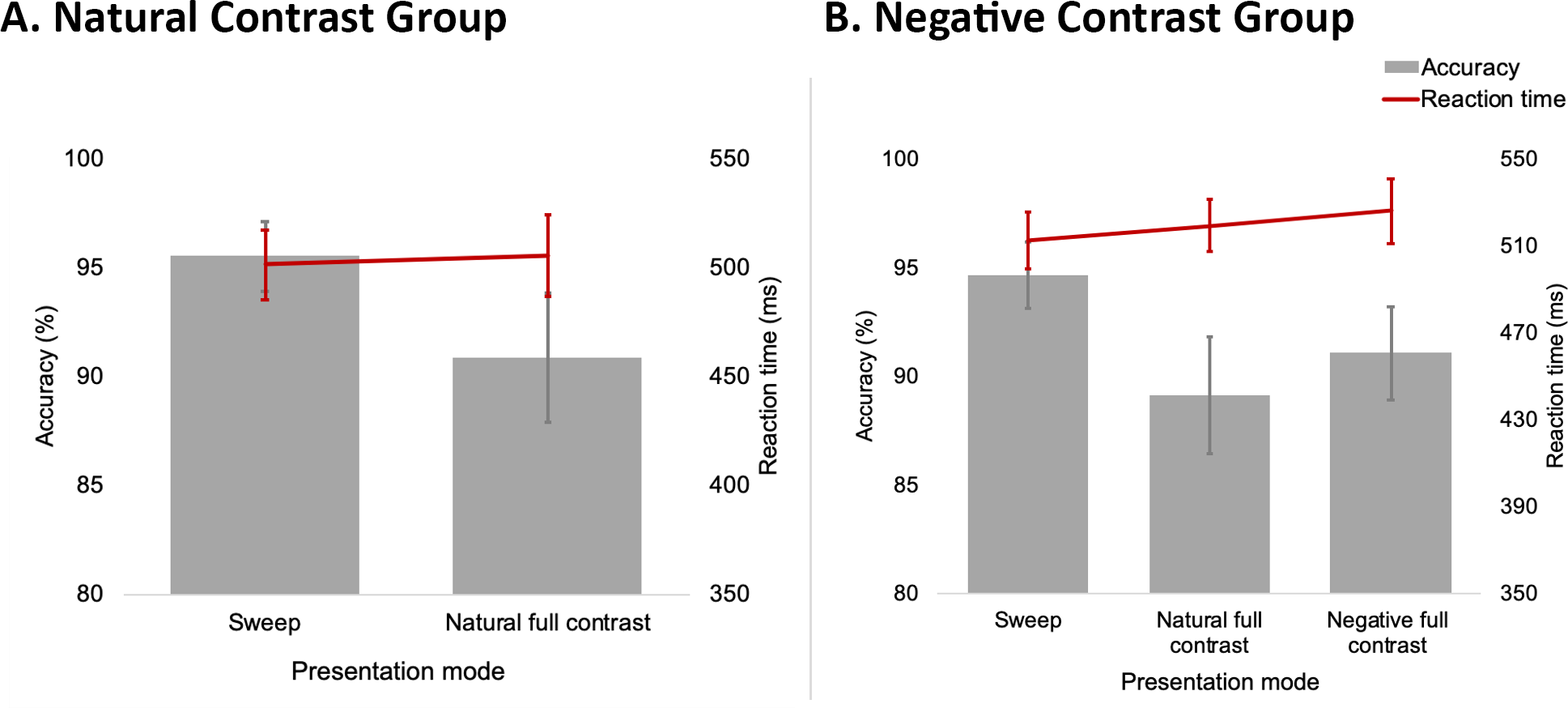

Supplement: Extended Data Figure 3-1 — Group-averaged accuracy and correct reaction times in the vigilance task during the sweep and full-contrast sequences, separately. A. Behavioral performance in the natural contrast group. B. Behavioral performance in the negative contrast group. These histograms show the means accuracy for each presentation mode. Secondary y-axis presents mean reaction time values (millisecond). Error bars represent 95% confidence intervals. Note that luminance and contrast were adjusted for figure visibility and are not representative of the actual (gamma-corrected) values. Download Figure 3-1, TIF file. [file enu-eN-NWR-0420-21-s02.tif]

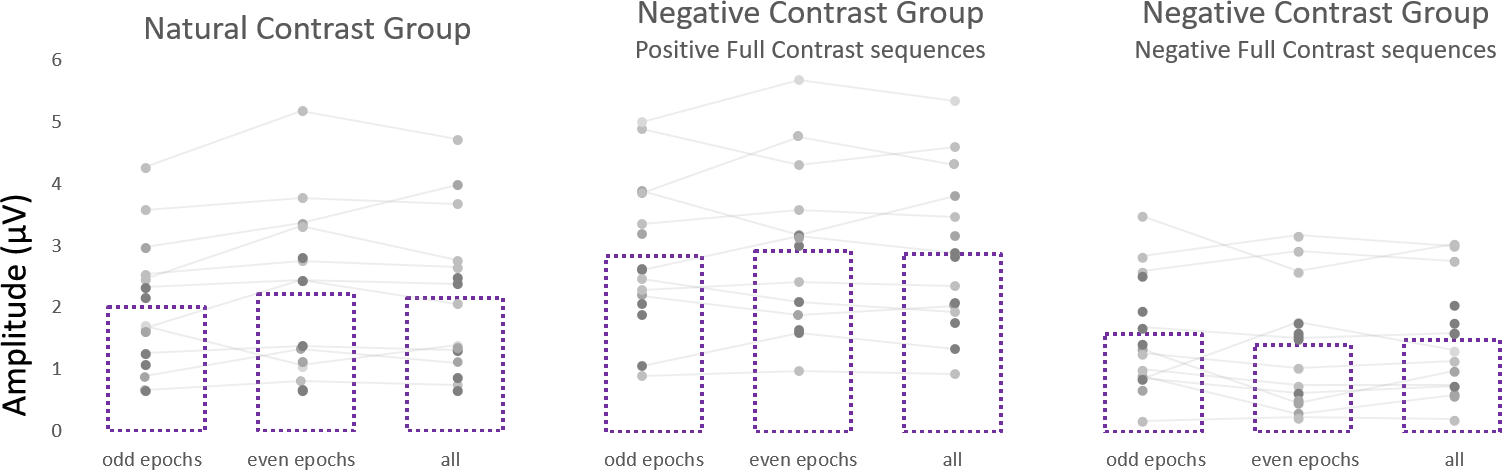

Supplement: Extended Data Figure 5-3 — Halving the number of full-contrast trials does not influence the amplitude of the face categorization response. The mean amplitude of the face categorization response to full-contrast stimulation sequences was stable whether based on all trials or only (odd/even) half of them, in natural and negative contrast groups (in individually defined ROIs; see Methods). Dots represent individual response amplitude values. The number of full contrast and sweep trials (n = 56 and n = 24, respectively) differed equally across groups; any potential SNR difference arising due to this difference of trial number should thus be similar across groups, and not contribute to our main findings. Nevertheless, we subsampled trials from the full-contrast condition in order to investigate whether the amplitude of the face categorization response was influenced by subsampling. To do so, we split full contrast trials into odd and even 'steps' and tested whether face categorization response amplitude was similar across sampling methods (odd, even, all trials). As shown in the figure below, face categorization response in full contrast sequences is relatively stable in amplitude irrespective of the number of trials. In a mixed ANOVA with group (natural contrast, negated contrast) and sampling method (odd, even, all) as factors, we confirmed that the sampling method did not influence the amplitude of the face categorization response significantly (F(1.37,38.39) = 2.69, p = .1, η2 = .09). Furthermore, the interaction between sampling method and group was non-significant (F(1.37,38.39) = .83, p = .4, η2 = .029). While null results should always be taken with caution, they suggest that the face categorization response we measured in full contrast trials across groups was stable whether the full sample of trials was used or if we halve it to get closer to the n of sweep trials. Download Figure 5-3, TIF file. [file enu-eN-NWR-0420-21-s05.tif]
