# Supplementary material for: Natural Contrast Statistics Facilitate Human Face Categorization
Source: eNeuro. 2022 Oct 4;9(5):ENEURO.0420-21.2022. doi: 10.1523/ENEURO.0420-21.2022 (PMC9536856; doi:10.1523/ENEURO.0420-21.2022)
Supplement: Extended Data Figure 5-1 — Individual ROIs in the Natural Contrast group. * = Participants which duplicate channels across general visual response ROIs and face categorisation ROIs. Download Figure 5-1, DOCX file. [file enu-eN-NWR-0420-21-s03.docx]

**Figure 5-1.** Individual ROIs in the Natural Contrast group. * = Participants which duplicate channels across general visual response ROIs and face categorisation ROIs.

|  | General visual response (12Hz) | | | |  | Face categorisation response (1.5Hz) | | | |
| --- | --- | --- | --- | --- | --- | --- | --- | --- | --- |
| S02* | Iz | O2 | Oiz | POI2 |  | P10 | PO10 | PO12 | POI2 |
| S03 | Oiz | Oz | POI1 | POOz |  | I2 | P10 | PO11 | PO12 |
| S04 | Iz | O2 | Oiz | Oz |  | P10 | PO10 | PO12 | PO7 |
| S05 | O1 | Oz | POO5 | POOz |  | P10 | PO10 | PO12 | PO9 |
| S06* | POO5 | POO6 | POOz | POz |  | O2 | Oz | PO9 | POO6 |
| S07* | I1 | Iz | Oiz | POI1 |  | I1 | P10 | PO12 | POI1 |
| S08 | Iz | Oiz | Oz | POOz |  | I2 | P10 | PO10 | PO12 |
| S09 | I1 | Iz | POI1 | POI2 |  | P10 | PO10 | PO12 | PO9 |
| S10* | O2 | PO10 | PO12 | POI2 |  | I2 | P10 | PO10 | PO12 |
| S11 | O2 | Oiz | POI1 | POI2 |  | P9 | PO10 | PO11 | PO9 |
| S12* | I2 | O2 | Oiz | POI2 |  | P10 | PO10 | PO12 | POI2 |
| S13 | O2 | PO10 | PO8 | POI2 |  | P9 | PO11 | PO7 | PO9 |
| S14 | O2 | Oiz | Oz | POO6 |  | P10 | PO10 | PO8 | PPO6 |
| S15 | O1 | Oz | POO5 | POOz |  | P10 | PO11 | PO7 | PO9 |
| S16 | I2 | Iz | Oiz | POI2 |  | PO11 | PO7 | PO9 | POI1 |
