# Supplementary material for: Natural Contrast Statistics Facilitate Human Face Categorization
Source: eNeuro. 2022 Oct 4;9(5):ENEURO.0420-21.2022. doi: 10.1523/ENEURO.0420-21.2022 (PMC9536856; doi:10.1523/ENEURO.0420-21.2022)
Supplement: Extended Data Figure 5-2 — Individual ROIs in the Negative Contrast group. * = Participants which duplicate channels across general visual response ROIs and face categorisation ROIs. Download Figure 5-2, DOCX file. [file enu-eN-NWR-0420-21-s04.docx]

**Figure 5-2.** Individual ROIs in the Negative Contrast group. * = Participants which duplicate channels across general visual response ROIs and face categorisation ROIs.

|  | General visual response (12Hz) | | | |  | Face categorisation response (1.5Hz) | | | |
| --- | --- | --- | --- | --- | --- | --- | --- | --- | --- |
| S01 | I1 | O1 | PO9 | POI1 |  | P10 | PO10 | PO11 | PO12 |
| S02 | Iz | Oiz | POI1 | POI2 |  | P10 | P9 | PO11 | PO12 |
| S03 | Iz | O2 | Oiz | Oz |  | P10 | PO10 | PO12 | PO8 |
| S04* | O1 | O2 | Oiz | Oz |  | O2 | PO10 | PO12 | PO8 |
| S05 | I1 | Iz | Oiz | POI1 |  | P10 | P8 | PO10 | PO12 |
| S06 | I1 | Iz | Oiz | Oz |  | P10 | PO10 | PO11 | PO12 |
| S07 | O2 | PO8 | POI1 | POI2 |  | Iz | P9 | PO11 | PO9 |
| S08 | O2 | Oz | POI2 | POO6 |  | P10 | P9 | PO11 | PO9 |
| S09 | O2 | Oiz | Oz | POI2 |  | P10 | P8 | PO10 | PO12 |
| S10 | O1 | Oiz | Oz | POI1 |  | P10 | P8 | PO10 | PO12 |
| S11 | O1 | Oiz | Oz | POOz |  | P9 | PO11 | PO12 | PO9 |
| S12 | Oz | POO5 | POOz | POz |  | P10 | P8 | PO10 | PO12 |
| S13* | I1 | I2 | POI1 | POI2 |  | I1 | I2 | PO12 | POI2 |
| S14 | I1 | O1 | Oiz | POI1 |  | P10 | PO10 | PO12 | PO9 |
| S15 | O1 | POI1 | POO5 | POO6 |  | P10 | P8 | PO10 | PO12 |
